# Description and Genome Characterization of Three Novel Fungal Strains Isolated from Mars 2020 Mission-Associated Spacecraft Assembly Facility Surfaces—Recommendations for Two New Genera and One Species

**DOI:** 10.3390/jof9010031

**Published:** 2022-12-23

**Authors:** Atul Munish Chander, Marcus de Melo Teixeira, Nitin K. Singh, Michael P. Williams, Anna C. Simpson, Namita Damle, Ceth W. Parker, Jason E. Stajich, Christopher E. Mason, Tamas Torok, Kasthuri Venkateswaran

**Affiliations:** 1 Biotechnology and Planetary Protection Group, Jet Propulsion Laboratory, California Institute of Technology, M/S 89-2, 4800 Oak Grove Dr., Pasadena, CA 91109, USA; 2 Pathogen and Microbiome Institute, Northern Arizona University, Flagstaff, AZ 86011, USA; 3 School of Medicine, University of Brasilia, Brasilia 70910-900, Brazil; 4 Department of Physiology and Biophysics and the WorldQuant Initiative for Quantitative Prediction, Weill Cornell Medicine, New York, NY 10021, USA; 5 Department of Microbiology and Plant Pathology, University of California—Riverside, Riverside, CA 92521, USA; 6 Lawrence Berkeley National Laboratory, Ecology Department, Berkeley, CA 94720, USA

**Keywords:** *Aaosphaeria pasadenensis*, *Floridaphiala radiotolerans*, fungi, genomics, Mars 2020 mission, *Pasadenomyces melaninifex*, phylogenetic analysis

## Abstract

National Aeronautics and Space Administration’s (NASA) spacecraft assembly facilities are monitored for the presence of any bacteria or fungi that might conceivably survive a transfer to an extraterrestrial environment. Fungi present a broad and diverse range of phenotypic and functional traits to adapt to extreme conditions, hence the detection of fungi and subsequent eradication of them are needed to prevent forward contamination for future NASA missions. During the construction and assembly for the Mars 2020 mission, three fungal strains with unique morphological and phylogenetic properties were isolated from spacecraft assembly facilities. The reconstruction of phylogenetic trees based on several gene loci (*ITS*, *LSU*, *SSU*, *RPB, TUB*, *TEF*1) using multi-locus sequence typing (MLST) and whole genome sequencing (WGS) analyses supported the hypothesis that these were novel species. Here we report the genus or species-level classification of these three novel strains via a polyphasic approach using phylogenetic analysis, colony and cell morphology, and comparative analysis of WGS. The strain FJI-L9-BK-P1 isolated from the Jet Propulsion Laboratory Spacecraft Assembly Facility (JPL-SAF) exhibited a putative phylogenetic relationship with the strain *Aaosphaeria arxii* CBS175.79 but showed distinct morphology and microscopic features. Another JPL-SAF strain, FJII-L3-CM-DR1, was phylogenetically distinct from members of the family *Trichomeriaceae* and exhibited morphologically different features from the genera *Lithohypha* and *Strelitziana.* The strain FKI-L1-BK-DR1 isolated from the Kennedy Space Center facility was identified as a member of *Dothideomycetes incertae sedis* and is closely related to the family *Kirschsteiniotheliaceae* according to a phylogenetic analysis. The polyphasic taxonomic approach supported the recommendation for establishing two novel genera and one novel species. The names *Aaosphaeria pasadenensis* (FJI-L9-BK-P1 = NRRL 64424 = DSM 114621), *Pasadenomyces melaninifex* (FJII-L3-CM-DR1 = NRRL 64433 = DSM 114623), and *Floridaphiala radiotolerans* (FKI-L1-BK-DR1 = NRRL 64434 = DSM 114624) are proposed as type species. Furthermore, resistance to ultraviolet-C and presence of specific biosynthetic gene cluster(s) coding for metabolically active compounds are unique to these strains.

## 1. Introduction 

The biological cleanliness of the National Aeronautics and Space Administration’s (NASA’s) mission-associated cleanrooms, where spacecraft are assembled and tested, is essential to meeting Planetary Protection (PP) requirements for continued robotic exploration of the solar system. Rigorous cleaning procedures are performed in these cleanrooms, including the Jet Propulsion Laboratory (JPL) Spacecraft Assembly Facility (SAF) and the Kennedy Space Center (KSC) Payload Hazardous Servicing Facility (PHSF) [1,2]. Nonetheless, after robust cleaning, viable microbial taxa have been detected in these facilities [3]. Extensive studies have been carried out on monitoring and characterizing the bacterial populations of these cleanrooms, specifically bacterial endospore-formers [1,4]. The presence of fungi in these facilities has been mostly ignored, despite the vast range of conditions that fungi can tolerate, including survival after exposure to various radiation conditions [5], and tolerance to radionuclide contaminants [6]. Fungal species are often reported to survive in extreme environments and in simulated Mars conditions [7]. Surfaces in these low-nutrient, temperature and moisture-controlled SAFs are considered extreme environments [3], and therefore, the characterization of fungal populations from these cleanrooms is important for assessing the risk of forward contamination. In addition, such extremophilic fungi might serve as model microorganisms for developing future cleaning and sterilization strategies. Understanding those traits that enable the survival and proliferation of fungi in extreme environments is important since they may compromise the science of future life-detection missions.

To provide meaningful information for current and future NASA PP biological monitoring, new microbial species discovered in cleanrooms need to be characterized for both phenotypic traits and functional genes that could result in survival in extreme environments. In addition, a new species must be placed within a traditional taxonomic framework so that it can be incorporated into future databases and detected in metagenomic data. For fungi, morphological, developmental, and physiological characteristics are the criteria upon which fungal taxonomy has traditionally relied [8]. Fungi present a broad and diverse range of phenotypic traits to adapt to various conditions, often within a single species [9]. Many fungal genes are cryptic; under normal laboratory conditions, the phenotypes of genetically distinct fungal species are impossible to differentiate, leading to misclassification. In addition, phenotypic divergence is often difficult to observe, particularly in filamentous fungi [8]. Thus, modern fungal taxonomy includes genealogical approaches such as single- or concatenated multi-gene phylogenies and whole genome sequence (WGS) based phylogenetic analysis, though the latter is limited due to the low number of fungal species for which genomes are publicly available. Novel species of fungi are now often detected via genealogical approaches rather than via differences in phenotypic characteristics [10].

Fungal genealogy presents its own challenges, as fungi are capable of genome rearrangement including changes in the number of chromosomes or parts of chromosomes, which allows them to adapt to adverse conditions much more quickly than would be possible via point mutations on stress-related genes [11]. Such genomic variation gives fungi an astounding level of biochemical complexity with a wide variety of strategies for interacting with their environments [12]. This phenotypic, genomic/biochemical complexity and plasticity present a challenge for fungal taxonomists when trying to accurately define fungal evolution. Unlike in bacteria, where most species are resolved by 16S ribosomal RNA-based phylogeny or by average nucleotide identity (ANI), multiple conserved genes are needed for accurate fungal taxonomic identification [13]. Hence, it is proposed that all three key features - morphology, genetics, and biochemistry be explored for correct fungal taxa determination [14].

Here, we present the characterization of three novel fungal strains from NASA spacecraft assembly facilities using this three-pronged approach. One of the objectives of this study was to define the phylogenetic novelty of three fungal strains, whose phylogenetic affiliations were not assigned to any known fungi. We used traditional fungal characterization via colony morphology and microscopy, and, in addition, performed multi-locus sequence typing (MLST) of up to six loci, internal transcribed spacer (*ITS)*, large subunit ribosomal ribonucleic acid (*LSU)*, small subunit ribosomal ribonucleic acid (*SSU*), RNA polymerase II gene (*RPB*), tubulin (*TUB)*, and translation elongation factor 1 alpha (*TEF*1). The WGS-based methods have increasingly been used to detect and characterize outbreaks for fungal pathogens [15,16,17], however, their efficacy on novel fungal strains is not well tested yet. Thus, the second objective was to clarify the phylogenetic relationship of the novel fungal genera with other closely related fungi using available WGS. The WGS-based taxonomic approach combined with MLST, morphological and microscopic characterizations enabled us to gain confidence in the description of these fungal taxa. Importantly, this article also evidenced the challenges in employing the WGS-based taxonomy approach to characterize novel fungal strains since limited WGS of fungal strains is available. As a third objective of this study, though not used for the taxonomy, bioactive compound production was predicted in these strains by characterizing biosynthetic gene cluster(s) (BGCs) from the WGS. 

In an ongoing microbial surveillance study of NASA’s Mars 2020 mission-associated environments, the polyphasic fungal taxonomy approach yielded a novel fungal species belonging to the genus *Parengyodontium* [18]. Continuing the effort, this is the first report on three novel fungal strains that were isolated from two NASA cleanroom facilities, two of which are well evidenced for belonging to novel genera and one as novel species.

## 2. Materials and Methods

### 2.1. Sample Collection and Isolation of Fungi

As previously reported, two NASA cleanroom facilities, JPL-SAF and KSC-PHSF, were sampled between April and September of 2018 [3]. JPL-SAF and KSC-PHSF are maintained to International Organization for Standards (ISO-7) certification requirements, which specify less than 352,000 >0.5-µm particles per m^3^ and 60 HEPA filtered air changes per hour [3]. In brief, JPL-SAF is vacuumed and then cleaned with Kleenol 30, a highly concentrated industrial strength cleaner and degreaser, which contains 12.5% ethylene glycol monobutyl ether, 1–5% nonylphenol ethoxylate, 1% dodecylbenzenesulfonate, and 1–4% silicic acid disodium salt. Daily cleaning at KSC-PHSF is performed by vacuuming and mopping with plain water. The air supply to the facilities is filtered through high-efficiency particulate air filters that trap >99% of 0.5-µm particles and above. At both JPL-SAF and KSC-PHSF, pre-moistened polyester 12” × 12” wipes (Sterile TexTra10 TX3225; Texwipe) were used to collect samples from cleanroom surfaces. The wipes were then placed in 500 mL bottles containing 200 mL of sterile phosphate-buffered saline (PBS) and shaken for 1 min. The volume of PBS suspension was reduced using a CP-150 InnovaPrep concentrating pipette (Innova Prep LLC) [19]. The *Aaosphaeria pasadenensis* FJI-L9-BK-P1 was isolated after spread plating the JPL sample on potato dextrose agar (PDA, Difco, #213400) containing chloramphenicol (25 µg/mL) and incubated at 25 °C for 7 days. The *Pasadenomyces melaninifex* FJII-L3-CM-DR1 was picked from spread plating a JPL sample on dichloran rose bengal chloramphenicol (DRBC) agar (Difco, # 258710) and grown at 25 °C for 7 days. Similarly, *Floridaphiala radiotolerans* FKI-L1-BK-DR1 was retrieved from a KSC-PHSF sample after spreading it on DRBC agar and incubating at 25 °C for 7 days. Irrespective of the isolation medium, subsequent growth of these strains was carried out on PDA medium unless an alternate culture medium is mentioned.

### 2.2. Morphological Analysis

For colony morphology characterization, the fungal strains were transferred to PDA and oatmeal agar (OMA, Difco, # 255210) and incubated at 25 °C. Colony diameter (in mm), structure, pigmentation, and other morphological characteristics were recorded after 21 days unless otherwise specified. Photographs of the colonies were taken using a smartphone camera. A slide cultivation technique [20] was used on PDA-grown cultures for cell morphology observations after 14–21 days. Briefly, a small block of agar was placed in the center of a sterile slide and all four sides of the agar were inoculated with the fungus. Subsequently, a sterile coverslip was gently placed on the top of the agar block. The slide culture was kept in a moist Petri dish lined with filter paper soaked in sterile water. After 14–21 days, the fungus grew out onto the coverslip and the slide. The cover slip was then gently removed with sterile forceps and placed on a clean microscopic slide with a drop of water or lactophenol cotton blue (Z68, Hardy Diagnostics) for microscopic observations. The light microscopy and phase contrast images were captured on an Olympus BX53 microscope with an Olympus DP25 camera and Olympus cellSens software. Differential interference contrast (DIC) images were generated on a Nikon Ti2-E inverted microscope with Nikon NIS Elements software. Cell morphological characters were measured with the Olympus cellSens or Nikon NIS Elements software.

### 2.3. Scanning Electron Microscopy

Sample preparation and scanning electron microscopy (SEM) were performed as described elsewhere [18]. In brief, fresh fungal samples were collected from cultures grown on PDA plates, immersed in chilled 2.5% glutaraldehyde (Ted Pella Inc., Redding, CA, USA) in 0.1 M sodium cacodylate buffer (Sigma–Aldrich, St. Louis, MO, USA), and incubated at 4 °C for 1 h. The cells were then washed three times in 0.1 M sodium cacodylate buffer followed by dehydration with isopropyl alcohol (IPA) via a series of incremental IPA concentrations from 50% to 100% (50%, 70%, 80%, 90%, 95%, and three times 100%). After the dehydration steps, the samples were stored at 4 °C in 100% IPA. As described previously [18], critical point drying was performed followed by SEM with an FEI Quanta 200F scanning electron microscope (Thermo Fisher, Waltham, MA, USA).

### 2.4. ITS-Based Fungal Identification

Initial identification of the fungi was performed by amplicon sequencing. Fungal biomass grown on PDA plates was collected and genomic DNA was extracted using a Maxwell-16 MDx automated system following the manufacturer’s instructions (Promega). The *ITS* region was amplified employing polymerase chain reaction (PCR), using primers *ITS* 1F (5′-CTT GGT CAT TTA GAG GAA GTA A-3′) [21], and Tw13 (5′-GGT CCG TGT TTC AAG ACG-3′) [22]. PCR conditions and sample preparation steps for sequencing were described elsewhere [23]. The *ITS* sequences were also included in the MLST-based analysis.

### 2.5. Whole Genome Sequence Generation

Genomic DNA was isolated from 1 g wet biomass scraped from the PDA plates, using the ZymoBIOMICS MagBead DNA kit (Zymo Research) and bead beating, followed by a Precellys homogenizer treatment. Quality of genomic DNA was verified by gel electrophoresis and quantified by spectrophotometric measurements (Nanophotometer NP80 Mobile, Implen). Library preparation followed as per the Illumina Nextera Flex Protocol (Illumina document # 1000000025416 v07). The initial amount of DNA for library preparation was quantified, and 5 to 12 cycles of PCR amplification were carried out to normalize the output depending on the input DNA concentration. The amplified genomic DNA fragments were indexed and pooled in a 384-plex configuration with dual-index adapters. WGS was performed on a NovaSeq 6000 S4 flow cell PE 2 × 150 platform with a paired-end module. The data were filtered with NGS QC Toolkit v2.3 for high-quality (HQ) vector and adaptor-free reads for genome assembly (cutoff read length for HQ 80%; cutoff quality score 20) [24]. The filtered reads were used for draft genome assembly with SPAdes v 3.15.4 [25] genome assembler (k-mer size: 32 to 72 bases), using default parameters. After initial phylogenetic classification, genomes for the three novel strains were reassembled using the Automatic Assembly for the Fungi (AAFTF v 0.3.3) pipeline [26], as follows: (i) illumina raw reads were trimmed using BBDuk module of BBMap (v 38.95) [27]; (ii) contaminant reads were removed using BBMap; (iii) the polished reads were assembled, using SPAdes v 3.15.4; (iv) contaminant contigs were identified and purged, using nucleotide Basic Local Alignment Search Tool (BLASTn) [28]; (v) duplicated contigs were identified and removed, using minimap2 [29]; (vi) assembled contigs were polished, using the Pilon v1.24 software [30]; (vii) the final scaffolds were sorted by length and the fasta headers renamed for deposit at the National Center for Biotechnology Information (NCBI). The assembly quality was verified with QUAST 5.1.0 [31]. 

### 2.6. MLST-Based Phylogenetic Analyses

We used the MLST approach for the phylogenetic affiliations of the three fungal strains. Initially, *ITS* amplicon sequences were used to determine preliminary genus assignment. After WGS, we extracted the desired DNA loci for MLST from each genome using a python wrapper script for NCBI BLASTn [32]; genomes were aligned to the UNITE database in the case of *ITS* sequences [33], or to a curated set of the desired genes deposited in Genbank from a variety of *Ascomycota* species. Once the target genes were located and extracted from each genome, we performed a BLASTn analysis against the NCBI nucleotide database to confirm the nearest gene identity [28], length, and initial taxonomical classification. The second round of BLASTn alignments and gene extractions were performed when BLASTn results of the pulled genes indicated a longer, better match to a more closely related species. Next, we used the Tree-Based Alignment Selector toolkit (T-BAS v2.1) for the phylogenetic placement of rDNA loci using the *Pezizomycotina* v2.1 database [34]. Subsequently, we performed a literature search on the MLST schemes for the taxonomic assessment of *Dothideomycetes* and *Chaetothyriales* using the following combination of loci: for *F. radiotolerans* and *P. melaninifex*: *ITS*, *LSU*, *TEF*1, *RPB*2, and *TUB,* and for *A. pasadenensis*: *ITS*, *LSU*, *TEF*1, and *RPB*2. Beyond the rDNA loci, we extracted the *TEF*1, *TUB*, and *RPB*2 loci from the three assembled genomes if not already extracted, using the BLASTn tool. Individual sequences from closely related *Dothideomycetes* and *Chaetothyriales* fungi were pulled out from the NCBI nucleotide database (Supplementary Tables S1–S3). Individual alignments for each gene were generated by practical alignments using the Sate and TrAnsitivity (PASTA) algorithm [35]. In smart-gap mode, we used ClipKIT to trim phylogenetically uninformative sites from the DNA alignments [36]. Phylogenetic trees were constructed from these alignments using the concatenation approach via IQ-TREE v 2.1.1 [37]. The best DNA substitution model was calculated with the ModelFinder approach [38], and branch support was inferred using ultrafast bootstraps and an SH-like approximate likelihood ratio test (SH-aLRT) [39]. The tree topologies were then visualized with FigTree v 1.4.4.

### 2.7. Genome Annotation

The assembled genomes were annotated using the funannotate v1.8 pipeline [40]. Initially, we identified and masked repetitive DNA content using TANTAN [41] and the funannotate mask command. Next, we predicted gene content using a series of evidence-based and *ab initio* gene prediction programs with the funannotate predict command. We used BUSCO to find conserved gene models for training the *ab initio* predictors Augustus [42], glimmerhmm [43], and snap [44]. We used the *chaetothyriales*_odb10 database for training the *P. melaninifex* genome, while the *dothideomycetes*_odb10 database was used for training both *A. pasadenensis* and *F. radiotolerans* genomes. We also used the self-training *ab initio* gene finder GeneMark-ES, using the option for fungal genomes in all three genomes. Next, all gene models were loaded into EVidenceModeler software to obtain a weighted consensus gene structure [45]; all predictors had weight = 1, but Augustus HiQ models were set to weight = 2. Gene models with less than 50 amino acids (aa) in length or identified as transposable elements were purged. tRNAs were detected using tRNAscan-SE [46]. Annotations were performed using the funannotate annotate function, applying previous functional analysis from Interproscan [47], Eggnog [48], Pfam [49], CAZYme [50], MEROPS [51], BUSCO [42], and Secreted proteins [52]. Gene Ontology (GO) terms were also appended to the final annotation file (.sqn). The assembled genomes were also submitted to fungiSMASH, the fungal-specific pipeline for antiSMASH [53], for prediction of the presence of biosynthetic gene clusters.

### 2.8. Whole Genome-Based Phylogenetic Tree 

To provide a broader overview of the phylogenetic placement of the two novel genera and one novel species, we constructed a phylogenomic tree based on conserved genes in fungi. For this purpose, we used the PHYling pipeline [54]. Initially, protein models representing the *Dothideomycetes* class (for phylogenomic analysis of *A. pasadenensis* and *F. radiotolerans*) or the *Chaetothyriales* order (for phylogenomic analysis of *P. melaninifex*) were downloaded from the Mycocosm portal [55]. Next, the HMMER. v3.3.2 hmmsearch tool was used to identify homologous sequences contained in the fungi_odb10 database which are used as a universal benchmark for evolutionary studies in fungi. Individual protein alignments were built by using the hmmbuild function of HMMER. v3.3.2 tool. Spurious positions were removed with the ClipKIT v 1.3.0 tool, using the smartgap function. Then, individual protein maximum-likelihood (ML) phylogenetic trees were generated using FastTree v 2.1.11 [56]. Next, we used ASTRAL v 5.7.1 to infer the species from gene trees, while accounting for gene tree discordance modeled by the multi-species coalescent method [57]. Finally, we used the species tree from ASTRAL software to correct the final topology, estimate branch lengths, and calculate the posterior probabilities for branch support using RAxML v8.2.12. The final tree was visualized with FigTree v 1.4.4. 

### 2.9. UV-C Exposure and Survival Evaluation

The evaluation of ultraviolet-C (UV-C) resistance in the novel fungal strains was carried out with minor modifications to the methods described previously [58,59]. High-grade aluminum coupons (Al 6061-T6) were precision cleaned for sterility as previously described [59]. Purified conidia were counted using a hemocytometer (Double Neubauer Counting Chamber, Hausser Scientific) after harvesting 40 days grown *A. pasadenensis* cultures at 25 °C on PDA. Conidial suspensions were diluted in sterile molecular biology grade water (Fisher Scientific) and ∼10^5^ conidia were added to each coupon followed by drying overnight at room temperature in a bio-hood. UV resistance of fungal hyphae/arthroconidia also deserves importance [60]. Conversely, for strains *P. melaninifex* and *F. radiotolerans*, three loopful of inoculum from around 30 days old culture were added in a 2 mL sterile microcentrifuge tube. The inoculum was crushed/chopped with sterile pipette tips and 500 μL of sterile molecular-grade water was added to the tube for making a suspension. The suspension was vortexed for 5 s and 50 μL of supernatant was seeded onto three aluminum coupons for each strain. The dried aluminum coupons were exposed at 1000 J/m^2^, 2000 J/m^2,^ and 3000 J/m^2^ of UV dose by using a UV Crosslinker CL-1000 (UVP Inc). The aluminum coupons were immersed directly into 5 mL potato dextrose broth (PDB, Difco) contained in a 15 mL sterile tube. The culture was observed for 7 days post-inoculation for any growth. The cultures showing growth on all three coupons were considered resistant to that specific dose of UV-C.

## 3. Results

A summary of genomic features and other annotations of the three draft genomes is presented in Table 1. The marker genes used to generate MLST were provided in Supplemental Tables S1–S3, annotations related to BGCs are in Supplemental Table S4, and proteome data used in developing the WGS-based phylogenomic tree are presented in Supplemental Table S5.

### 3.1. Taxonomy and Description of the Strain A. pasadenensis

Type species: *Aaosphaeria pasadenensis* Venkateswaran & Chander sp. nov. - MycoBank Number: 844547. The species name, pa.sa.de.nen.sis N.L., is designated to the city of Pasadena in California, where the JPL is located and isolated the strain.

Holotype: USA: Pasadena, CA, 34.1478° N, 118.1445° W, JPL-SAF cleanroom floor, where Mars 2020 mission components were assembled, isolated on April 17, 2018, Kasthuri Venkateswaran. Holotype is stored in a metabolically inactive state as a lyophilized culture at the United States Department of Agriculture (USDA), Northern Regional Research Laboratory (NRRL), Agricultural Research Service (ARS), USA; ex-holotype culture, FJI-L9-BK-P1 = NRRL 64424 and at the Deutsche Sammlung von Mikroorganismen und Zellkulturen (DSM 114621).

Diagnosis: A monotypic genus in the family *Roussoellaceae*, order *Pleosporales, Dothideomycetes,* and *Ascomycota*. Filamentous, fast growing, anamorphic fungus, for which recognizable sexual morph was not observed. At an early stage (25–30 days old culture), round bulb-shaped conidia are formed by a flattened conidiogenous cell and released. Mature colonies include dark brown to black conidiomata releasing oval shaped dark brown to black conidia. The released conidia remain attached in bunches at termini of the elongated sac-like conidiophore. Thus, *A. pasadenensis* is morphologically distinguished from members of the family *Roussoellaceae* by having these different shapes, forms, origin of two different kinds of conidia, which are released by morphologically different conidiophores/conidiogenous cells [formation of conidiogenous cell producing round conidia (probably produced once only)] and elongated sac-like conidiophore originating from mycelial walls that produces several conidia). The forms of conidial fusion via the formation of short and thin conidial anastomosis tubes (CAT) are unique to the strain *A. pasadenensis*. 

**Figure 1:**
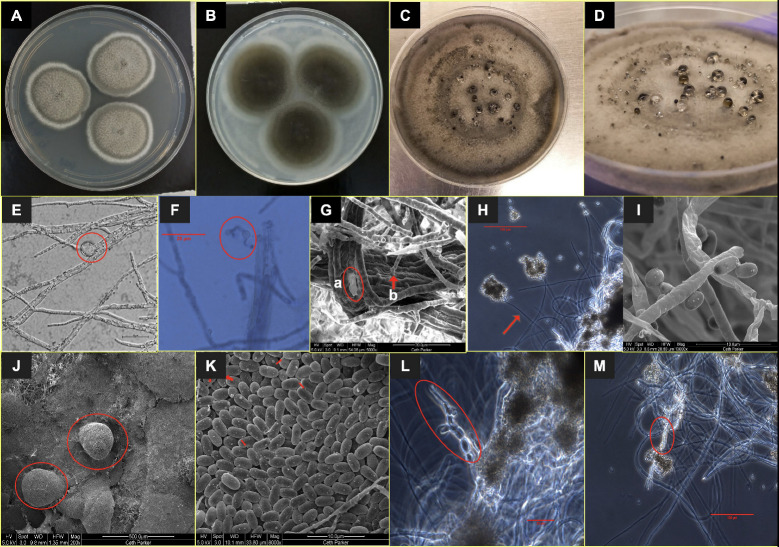
Colony and cell morphology of *A. pasadenensis*. Colony surface of *A. pasadenensis* after 8 days of incubation at room temperature (25 °C) on (**A**) PDA medium and (**B**) OMA medium. (**C**, **D**): Round, immersed, brown to black conidiomata on an aged PDA plate. (**E**, **F**): Early age liberated conidia that are germinating. (**G**): Conidiogenous cells, encircled (**H**, **I**): Late-stage elongated conidiophore (arrow) and conidia. (**J**): Conidiomata. (**K**): Conidia and red markups showing CATs. (**L**, **M**): Arthroconidia.

Description: *A. pasadenensis* is a fast-growing filamentous fungus. On day 8, the colonies on PDA and OMA are round at 36 mm and 46 mm in diameter, respectively, and have greenish-brown centric rings surrounded by young white hyphae (Figure 1A,B). The mature 2-month-old culture on PDA plates is brownish-black due to conidiomata (Figure 1C,D). Two types of conidia are formed. In 25–30-day old cultures on PDA, the early conidia are produced at the apical end of solitary or branching vegetative hyphae and are spherical (Figure 1E,F). Conidiogenous hyphae seem to release these conidia from a spherical formation at the terminal part of the hypha/conidiogenous cell (Figure 1E,F). The apical portion of conidiogenous hyphae following the release of conidia is flattened, irregular, and appears like a flaccid sac ~19 µm long (Figure 1G(a)). Formation of conidiomata is observed in two-month-old PDA cultures and the late conidia are part of conidiomata. Ovoid conidia are ~3 × 1.8 µm (Figure 1I) and are produced one by one from a short or elongated sac-like conidiophore originating from mycelial walls (Figure 1G(b),H,I). The conidiophores are aseptate and release conidia along with mucus slime, in which conidia are trapped agglomerating in bunches of hundreds (Figure 1K–M). The brownish-black conidia are trapped in the mucus exudate (Figure 1C,D) with dark black spots observed on the PDA plate (Figure 1D). Conidiomata are brown to black, erumpent, pear-shaped, and ~250 µm × 220 µm (Figure 1C,D,J). The conidia are observed to communicate with each other via short and thin CATs in SEM images (Figure 1K). Deep in the center of the colony, arthroconidial structures are observed under the phase contrast microscope (Figure 1L,M). The strain is resistant to UV-C doses of up to 2000 J/m^2^.

Ecology/Substrate/Host: The strain was isolated from the cleanroom floor at JPL-SAF.

Genomic characteristics: The estimated genome size of *A. pasadenensis* is 38.78 Mb. The GC content is 49.73%. The draft genome was assembled from 122 contigs (N50 = 943,274, L50 = 14) and the raw sequence, as well as the draft genome, were submitted to the NCBI database (Accession#: JAKLMB000000000). A total of 12,248 genes were identified and further classified into 12,141 mRNAs and 107 tRNAs. A total of 2295 GO and 11,235 Eggnog terms, 3140 Interpro and 8359 Pfam domains, 588 CAZYmes, 395 MEROPS peptidases, 1204 secreted proteins, and 3682 BUSCO orthologs were annotated. The BGC analysis and predicted gene clusters are presented in Supplemental Table S4. The fungiSMASH pipeline predicted 30 gene clusters potentially capable of expressing secondary-metabolite-forming enzymes, including 11 non-ribosomal peptide synthase (NRPS) or NRPS-like components, 12 type-1 polyketide synthases (T1PKS), and five terpene compounds, with additional clusters, predicted to use multiple mechanisms of secondary metabolite synthesis. Three predicted BGCs within the genome of *A. pasadenensis* exhibited close matches to known clusters, including BGCs for the synthesis of the siderophore and virulence promoter dimethyl coprogen [61], the anti-tumor compound clavaric acid [62], and melanin [63]. 

**Figure 2:**
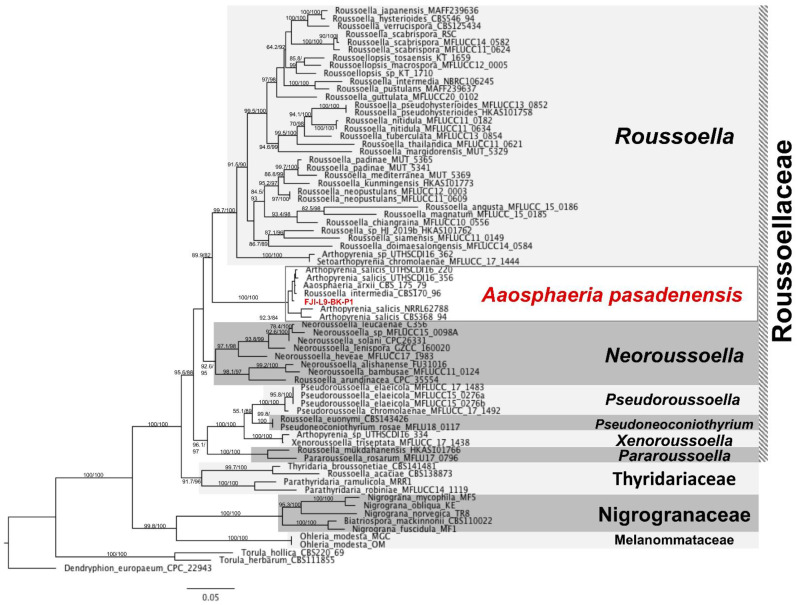
The MLST of *A. pasadenensis*. The genes *ITS, LSU, RPB2, TEF*1 were used to investigate the phylogenetic placement of the *A. pasadenensis* via ML tree on the IQTREE2 software. The branches are proportional to the number of mutations and 1000 ultrafast bootstraps and SH-aLRT was used to test branch support and were added to each corresponding branch of the tree. The tree was rooted with *Dendryphion europaeum* CPC 22943.

Phylogenetic placement of the strain *A. pasadenensis*: According to phylogenetic analysis based on the *ITS, LSU, TEF1*, and *RPB2* loci, the strain FJI-L9-BK-P1 is placed within the *Roussoellaceae* family, order *Pleosporales*, class *Dothideomycetes* (Figure 2). *Aaosphaeria arxii* CBS 175.79 is a member of *Roussoellaceae* since this is placed on a very close branch to the genera *Roussoella, Neoroussoella, Pseudoroussoella, Pseudoneoconiothyrium, Xenoroussoella*, and *Pararoussoella*. *Aaosphaeria* previously has been placed incorrectly in *Dacampiaceae* [64].

**Figure 3:**
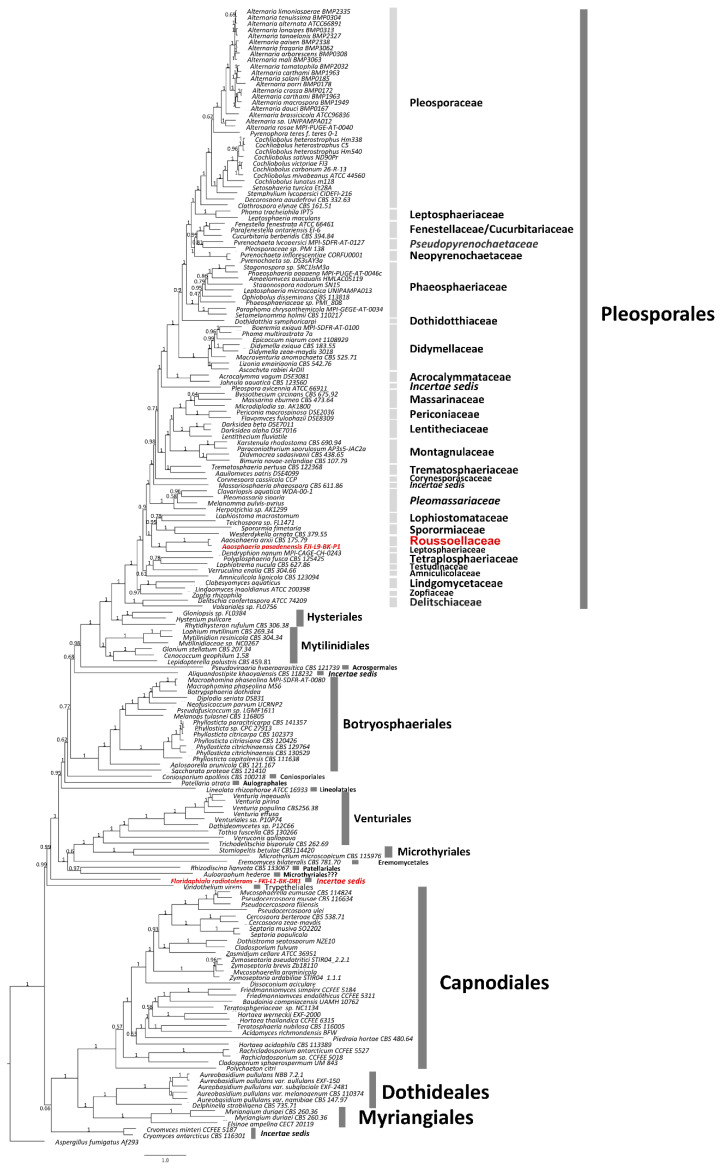
The WGS-based phylogenomic analysis for *A. pasadenensis* and *F. radiotolerans*. A phylogenomic tree was constructed for two strains *A. pasadenensis* and *F. radiotolerans*. ML tree was constructed using the RAxML and ASTRAL software. *Aspergillus fumigatus* Af293 was set as the outgroup and the branches are proportional to the number of mutations. Branch fidelity used posterior probabilities, which were added next to the corresponding branches.

Our phylogenomic analysis suggests that *Roussoellaceae* is closely related to *Lophiostomataceae, Sporormiaceae,* and *Leptosphaeriaceae,* and this is in agreement with the MLST-based phylogenetic analysis (Figure 3). More specifically, the species, *A. pasadenensis*, is a sister group of *Roussoella* and forms a triad along with the genus *Neoroussoella.* In this clade, some strains cluster along with FJI-L9-BK-P1, such as *Arthopyrenia salicis* UTHSC: DI16-220, UTHSC:DI16-356, NRRL 62788, and CBS 368.94, *Aaosphaeria arxii* CBS175.79, and *Roussoella intermedia* CBS 170.96. However, these strains are taxonomically misplaced/misidentified for many reasons: (i) The genus *Arthopyrenia stricto sensu* is composed of two species, *A. fallaciosa* and *A. cerasi* [65], and those are members of the order *Trypetheliales,* while the species *A. salicis* is placed within the order *Pleosporales.* This was also confirmed by our phylogenomic analysis (Figure 3). *A. salicis* was initially described by Massalongo in 1852 but differs from *Arthopyrenia stricto sensu* [66]. Thus, *Arthopyrenia* is polyphyletic and, therefore, the nomenclature should be amended. (ii) The strain *Roussoella intermedia* CBS 170.96 clusters in the same branch as *A. pasadenensis* but *R. intermedia* CBS 170.96 is misidentified since it does not belong to *Roussoella* genus, as per the MLST analysis performed in this study. The species *A. arxii* CBS 175.79 was initially described by Aptroot in 1995 and is composed of a single species [67]. The morphological and microscopic characteristics of *A. arxii* described by Aptroot in 1995 do not match with FJI-L9-BK-P1. Moreover, even though strains CBS 175.79 and *A. pasadenensis* are related, our detailed phylogenomic analysis suggests that they are distinct (Figure 3).

### 3.2. Taxonomy and Description of the P. melaninifex 

*Pasadenomyces* Venkateswaran, Chander & Singh, gen. nov. - Mycobank No: 844474.

Etymology: Pa.sa.dee.no.my.sees N.L. named after the city of Pasadena in California, USA, where the fungus was discovered. 

Description: Monotypic genus to accommodate a novel fungal species in the family *Trichomeriaceae*, order *Chaetothyriales, Eurotiomycetes,* and *Ascomycota*. Filamentous, slow-growing, anamorphic fungus. Recognizable sexual morph is absent. Septate hyphae. Prior to fragmentation to arthroconidia, cells are swollen and dumbbell-shaped, which is unique to this genus and is not reported in any phylogenetically close relatives. New arthroconidia originate either from old swollen arthroconidial hyphae or from a node-like structure with blastic proliferation.

Type species: *Pasadenomyces melaninifex* Venkateswaran, Chander & Singh sp. nov.-MycoBank Number: 844548. The species name, me.la.ni.ni.fex N.L., is given due to the black coloration of the fungal colony, which is likely due to melanin production.

Holotype: USA: Pasadena, CA, 34.1478° N, 118.1445° W, JPL-SAF cleanroom floor, where Mars 2020 mission components were assembled, isolated on September 25, 2018, Kasthuri Venkateswaran. Holotype is stored in a metabolically inactive state as a lyophilized culture at the USDA NRRL, ARS, USA; ex-holotype culture, FJII-L3-CM-DR1 = NRRL 64433 and Deutsche Sammlung von Mikroorganismen und Zellkulturen (DSM 114623).

Diagnosis: Based on morphological characteristics, *P. melaninifex* is related to *Lithohypha guttulata,* but phylogenetically forming a distant clade and morphologically distinguished from it by its longer branches, the frequent presence of swollen dumbbell-shaped structures that after separation give rise to mature arthroconidia, and pear-shaped cells at the apex of mature hyphae. In addition, the presence of round conidia is observed which is not reported in *L. guttulata* and other neighboring members. Arthroconidia, when separated, leave behind blunt-ended “birth scars.” The presence of conidia and their germination is observed. In addition, globose cells reported in *L. guttulate* were not observed in *P. melaninifex.*

**Figure 4:**
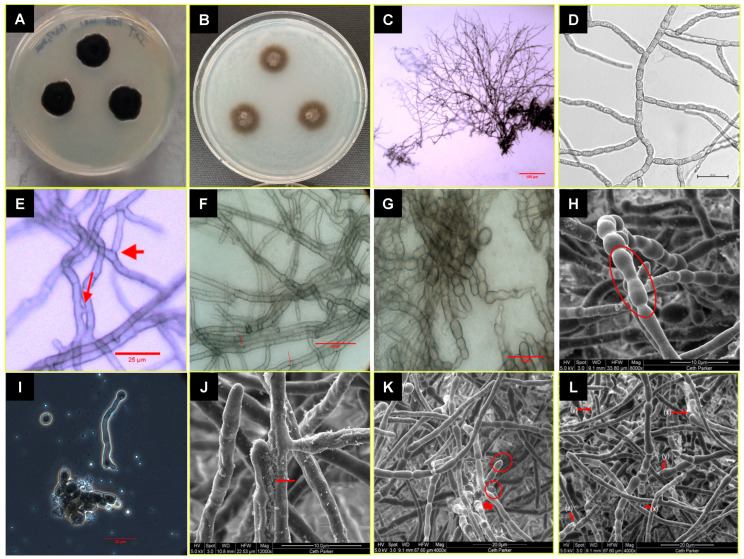
Colony and cell morphology of *P. melaninifex*. Colony morphology on day 21 of incubation at room temperature (25 °C) on (**A**) PDA medium and (**B**) OMA medium. (**C**): Blastic proliferation of hyphae. (**D**): Arthroconidia form readily and branch at roughly uniform angles. (**E**, **F**): Hyphael anastomosis in young vegetative hyphae. (**G**): Mature hyphae. (**H**): Arthroconidia and dumbbell-shaped hyphae. (**I**): Conidia on the top left corner; germinated conidia with long tube formation in the center of the image and a clump of arthroconidia in the lower center of the image, image is taken at 100x. (**J**): Hyphael anastomosis. (**K**): Arthroconidia radiating out from the arthroconidial node (arrow), arthroconidial blunt ends are encircled. (**L**): L_(x)_ and L_(y)_ shows hyphael anastomosis; L_(z)_ represents arthroconidial breaks.

Description: On day 21, the fungal colonies on PDA are 19 mm in diameter, black colored, slightly brown shaded, velvety, and ovoid with irregularities (Figure 4A). Colonies on OMA are 18 mm in diameter, gray-brownish and diffuse, rough-textured at center with brown cottony exudates (Figure 4B). The young vegetative hyphae are smooth walled, branched, septate, hyaline, composed of oblong cells (Figure 4E,F). Hyphae show blastic proliferation (Figure 4C,D,K), thus, branched at more regular angles. Hyphal anastomosis is observed in young hyphae (Figure 4E,F). Around two months post-inoculation, the fungus forms swollen, dumbbell-shaped structures prior to fragmentation to arthroconidia (~17–19 µm x 2.5 µm in dimension; Figure 4H,K,L). In addition, the presence of round conidia is observed in crushed culture inoculum which is not reported in *L. guttulata* and other members (Figure 4I). Smooth-walled hyphae and arthroconidia are observed, fused at instances via CAT-like tubes (Figure 4E,F,L marked by red arrows, y). Hyphal and anthrocondial fusion also seems to be facilitated by cottony cement-like meshwork (Figure 4I,J,L). Laterally aligned anthroconidia also show fusion (Figure 4J,L, red arrows marked as x). Crowding arthroconidia in light microscopic images (Figure 4C,G) appear as a sub-colony on the PDA plate (Figure 4B). The strain is resistant to UV-C doses of up to 3000 J/m^2^.

Ecology/Substrate/Host: Isolated from the JPL-SAF cleanroom floor, where Mars 2020 spacecraft components were assembled. 

Genomic characteristics: The estimated genome size of *P. melaninifex* FJII-L3-CM-DR1 is 27.60 Mb with a GC content of 48.13%. The genome was assembled from 972 contigs (N50 = 498,971, L50 = 17). The raw sequence, as well as the draft genome were submitted to the NCBI database (Accession#: JAKLMI000000000). We identified 10,961 genes that are divided into 10,856 mRNAs and 105 tRNAs, and annotated 2107 GO and 9,309 Eggnog terms, 2,968 Interpro and 7,070 Pfam domains, 297 CAZYmes, 346 MEROPS peptidases, 604 secreted proteins, and 2,928 BUSCO orthologs. Eleven BGCs were also identified in the FJII-L3-CM-DR1 genome, including 5 NRPSs, 3 T1PKS, 2 terpenes, and one combination of NRPS and T1PKS. Strong (100%) matches to known BGCs include those for melanin, the radical-scavenging compound pyranonigrin E [68], and the industrial pigment monascorubrin [69] (Supplemental Table S4).

**Figure 5:**
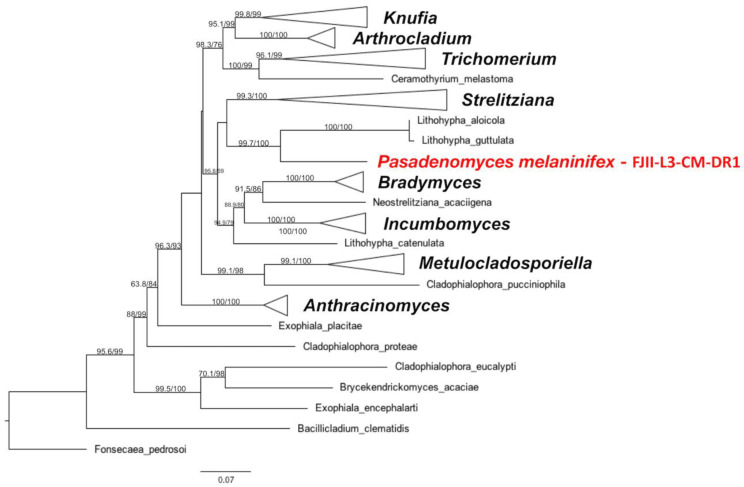
The MLST for the strain of *P. melaninifex*. Genes *ITS*, *LSU*, *TEF*1, *RPB*1, and *TUB* were used to investigate the phylogenetic placement of *P. melaninifex* via ML tree on the IQTREE2 software. The branches are proportional to the number of mutations and 1000 ultrafast bootstraps and SH-aLRT was used to test branch support and added to each corresponding branch of the tree. The tree was rooted with *Fonsecaea pedrosoi*.

**Figure 6:**
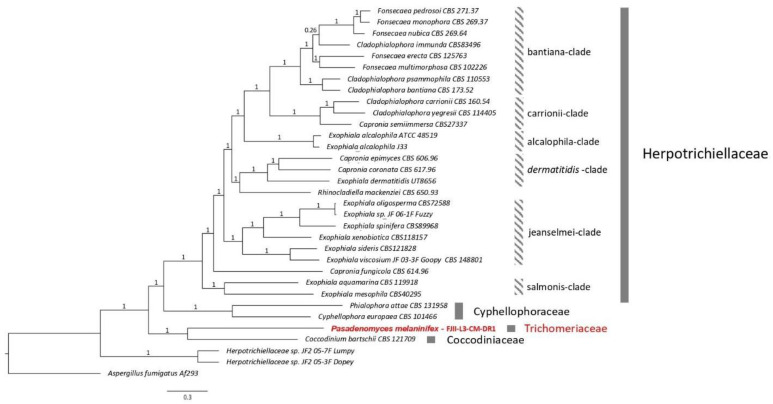
WGS-based phylogenomic analysis for *P. melaninifex*. ML tree among nine *Cordycipitaceae* fungi using the RAxML and ASTRAL software. *A. fumigatus* Af293 was set as the outgroup and the branches are proportional to the number of mutations. Branch fidelity used posterior probabilities, which were added next to the corresponding branches.

Phylogenetic placement of the *P. melaninifex*: An initial assessment of *P. melaninifex* based on *ITS* region sequencing suggested a genetic similarity to taxa in the family *Trichomeriaceae*, order *Chaetothyriales*. MLST analysis based on the *ITS*, *LSU*, *TEF*1, *RPB*2, and *TUB* markers revealed that this strain is a sister species to *Lithophila aloicola* and *L. guttulata* (Figure 5). *P. melaninifex* was thus confirmed to be a member of the family *Trichomeriaceae.* According to the WGS-based phylogenomic analysis, *P. melaninifex* was placed in a more basal position of the tree and is more closely related to *Coccodiniaceae* and *Cyphellophoraceae* (Figure 6).

### 3.3. Taxonomy and Characteristics of F. radiotolerans 

*Floridaphiala* Venkateswaran, Chander & Singh, gen. *nov*.- Mycobank No: 844477.

Etymology: Flo.ri.da.phi.a.la N.L. The recommended new genus is named after the state of Florida, USA. KSC is situated in Cape Canaveral, Florida, where the sample was collected. 

Description: Monotypic genus to accommodate a novel fungal species such as *Dothideomycetes incertae sedis*, *Dothideomycetes,* and *Ascomycota.* Slow-growing, anamorphic fungus, the recognizable sexual morph is absent. Vegetative hyphae are septate. Mature hyphae occasionally form round concatenated prominently enlarged chlamydoconidium-like cells with cantaloupe-resembling surfaces. Anastomosis enables the interaction of various round cell types and clump formation.

Type species: *F. radiotolerans* Venkateswaran, Chander & Singh, sp. nov. - MycoBank number: 844549. The novel species name, ra.dio.to.le.rans N.L., reflects the strain’s resistance to ultraviolet radiation.

Holotype: USA: Cape Canaveral, FL, 28.5729° N, 80.6490° W, SAF cleanroom floor, where Mars 2020 mission components were finally assembled before launch. Isolation on 12 June 2018, Kasthuri Venkateswaran. The holotype is stored in a metabolically inactive state as a lyophilized culture at the USDA NRRL, ARS, USA; ex-holotype culture, FKI-L1-BK-DR1 = NRRL 64434 and Deutsche Sammlung von Mikroorganismen und Zellkulturen (DSM 114624).

Diagnosis: Based on colony morphology, *F. radiotolerans* is related to *Spissiomyces aggregatus,* but phylogenetically forming a distant clade. Microscopic characteristics distinguish it from other *Dothideomycetes* fungi in terms of chlamydoconidium-like arrangements, their structure, and ornamentation that are unique to the genus. Chlamydoconidia formation is one of the morphological characteristics of *Candida albicans* [70]. In *Candida albicans*, chlamydoconidia are usually seen at the tips of hyphae, whereas in *F. radiotolerans,* cells similar to chlamydoconidia are found not only at the tips of hyphae but also intercalary in positions. 

**Figure 7:**
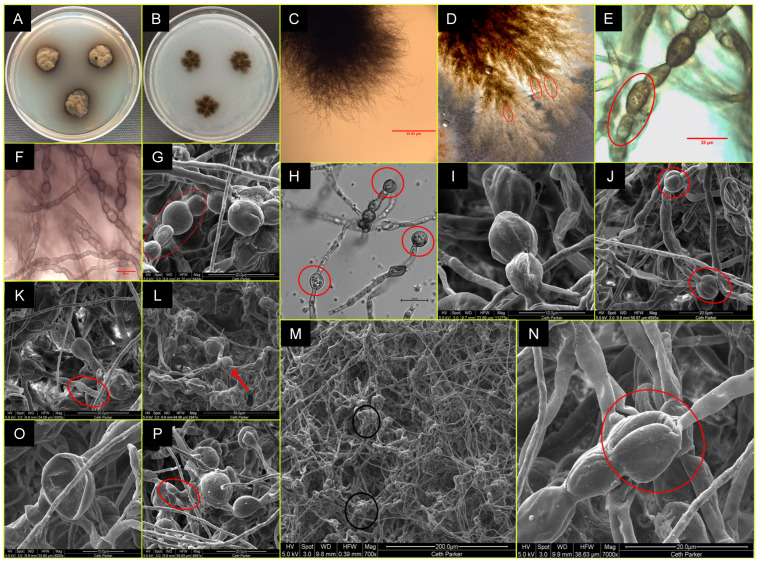
Colony and cell morphology of *F. radiotolerans*. Colony surface of *F. radiotolerans* after 14 days of incubation at room temperature (25 °C) on (**A**) PDA medium and (**B**) OMA medium. (**C**): Bush form vegetative morphology of young vegetative hyphae. (**D**): Highly branched mature hyphae showing dark brown nodes of conidiomata. (**E**–**F**): Vegetative hyphae are smooth-walled, septate with chlamydoconidium-like cells. (**G**): Chlamydoconidium-like cells. (**H**): DIC microscopy showing mature bulbous swollen chlamydoconidium-like cells containing endoconidium-like round structures. (**I**): Chains and aggregated form of chlamydoconidium-like cells. (**J**): Chlamydoconidium-like cells also exist solitary (**K**): Germinating new hyphae emerging out from a chlamydoconidium-like cell. (**L**): Hatched chlamydoconidium-like cell giving rise to multiple hyphal outgrowths. (**M**): clumping/anastomosis of chlamydoconidium-like cells. (**N**–**O**): Rupture/shrinkage of chlamydoconidium-like cells. (**P**): Formation of new young hyphae coming out from chlamydoconidium-like cells.

Description: After 14 days of incubation at 25 °C, the colonies on PDA (16 mm) and OMA (14 mm) are light brown to black, irregular in shape, raised, and diffused (Figure 7A,B). Vegetative hyphae are septate and branched. Chlamydoconidium-like cells form at intercalary and apical hyphal positions at 21–30 days aged fungal colonies (Figure 7E,F,H). These cells are often septate and approximately 18 µm × 11 µm in diameter. (Figure 6E; cells marked by a red circle), are globose and ovoid to pear-shaped, typically 10 µm–15 µm in diameter. (Figure 7E–J). SEM images confirm the surface ornamentation of swollen cells with cantaloupe-resembling ridges that gain more surface structure over time (Figure 7I–K,N). The catenated chlamydoconidium-like swollen cells seem to interact with regular hyphae or appear to be the origination source for new hypha (Figure 7J,K,M,O). Interactions between various cell types and occasional clumping of swollen cells are observed in *F. radiotolerans* (Figure 7K,M,N). Clumped chlamydoconidium-like cells may be the reason for dark black dot appearances on the colonies that are visible with the naked eye (Figure 7A,D). Shrinkage and rapture of swollen cells were also observed over time (Figure 7H,L,N–P). The strain is resistant to UV-C dose of up to 3000 J/m^2^.

Ecology/Substrate/Host: The strain was isolated from the cleanroom floor at KSC-PHSF where Mars 2020 mission spacecraft was checked for launching.

Genomic characteristics: The estimated genome size of *F. radiotolerans* is 24.89 Mb with a GC content of 52.18%. The genome was assembled from 109 contigs (N50 = 169,985, L50 = 17). The raw sequence, as well as the draft genome were submitted in NCBI (Accession#: JAKLMZ000000000). We identified 8582 genes that are divided into 8505 mRNAs and 77 tRNAs. We annotated 2099 GO and 7651 Eggnog terms, 2911 Interpro and 5866 Pfam domains, 266 CAZYmes, 257 MEROPS peptidases, 578 secreted proteins, and 3412 BUSCO orthologs. Twelve biosynthetic gene clusters were predicted within the genome of *F. radiotolerans*, including 3 NRPS/NRPS-like clusters, 6 T1PKS, and 3 terpene clusters (Supplemental Table S4). The only gene cluster with a 100% match to known BGCs is that of melanin.

**Figure 8:**
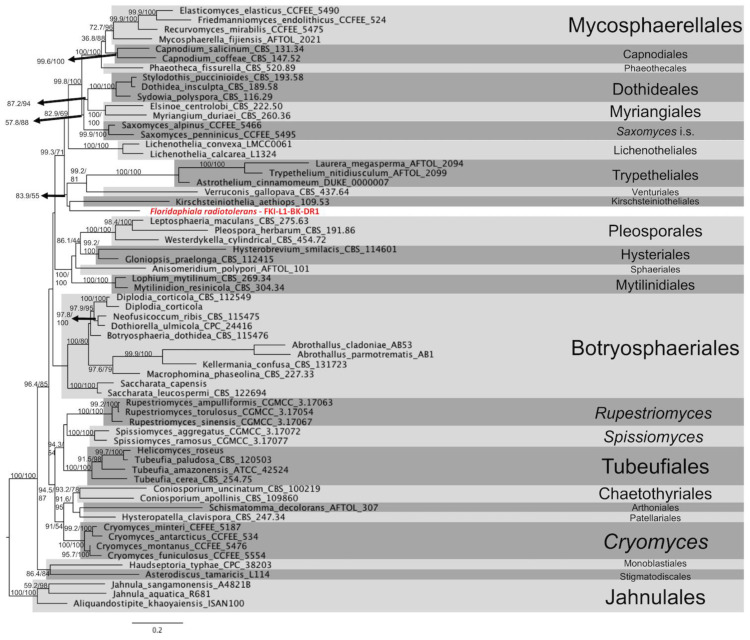
The MLST tree of *F. radiotolerans*. Genes *ITS*, *LSU*, *TEF*1, *RPB*2, and *TUB* were used to investigate the phylogenetic placement of the *F. radiotolerans* via ML tree on the IQTREE2 software. The branches are proportional to the number of mutations and 1000 ultrafast bootstraps and SH-aLRT was used to test branch support and added to each corresponding branch of the tree. The tree was rooted with members of the order *Jahnulales*.

Phylogenetic placement of *F. radiotolerans*: An initial assessment based on *ITS* amplicon sequencing was inconclusive due to low blast hit scores and less than 90% match to any described taxa in the NCBI database. However, the *ITS* sequences suggested that this fungus belonged to the class of *Dothideomycetes*. Therefore, we used an MLST scheme *ITS*, *LSU*, *TEF*1, *RPB*2, and *TUB* to better understand its phylogenetic position within the fungal order from this particular class and found that the species mapped next to *Kirschsteiniothelia aethiops* belonging to the order *Kirschsteiniotheliales* (Figure 8). However, this split is compromised by moderate aLRT and bootstrap support (83.9/55), suggesting that *F. radiotolerans* has its own evolutionary trajectory within the class of *Dothideomycetes.* The phylogenomic analysis also suggests a unique phylogenetic pattern for this fungus (Figure 3). The species *F. radiotolerans* is mapped between *Microthyriales* and *Trypetheliales,* and this possibly indicates that it may constitute a novel class/family within *Dothideomycetes.* For now, we have a more conservative nomenclature and defined this as an *incertae sedis*.

## 4. Discussion

Quantitative and qualitative estimation of NASA’s cleanroom bioburden is critically important to its Planetary Protection goals and human exploration missions [3,71]. NASA’s Planetary Protection program aims to prevent forward contamination in its robotic missions to other planets during space exploration. Therefore, identification and prediction of functional properties of microbial species present on spacecraft surfaces and associated cleanroom facilities are crucial in developing technologies to eradicate them. As part of this program, cataloging fungal species and determining their genotypic and phenotypic characteristics would allow NASA to improve cleaning strategies, thus avoiding forward contamination, and enabling uncompromised scientific data collection for future life-detection missions.

Molecular sequencing technologies and in particular genomic sequencing have become essential for microbial identification [13]. For example, two strains originally identified as *Aspergillus parasiticus* in NCBI were reclassified as *A. flavus* based on the genome sequence [72]. Previous to broad access to WGS [14], the *ITS* region sequence was the primary fungal barcode for the identification of uncharacterized fungal strains (without any *a priori* knowledge), but there are many genera for which the *ITS* region does not provide enough resolution for species identification [13]. Subsequently, researchers started using protein-coding genes (e.g., *TEF*1, *BenA*, *CaM*, *TUB*, etc.) for fungal identification under the phylogenetic species concepts. However, standardization was lacking [73,74,75], and no cutoff scores for fungal species identification were available [76]. Furthermore, ANI analysis is not regularly used for the identification of fungi as practiced for bacterial species because fungal genomes are complex and their WGS are scarce in public databases [77]. Hence, whenever possible, MLST and WGS-based analyses are employed to reconstruct species trees and to determine the closest relatives of an unknown fungal strain [13,78,79]. In this study, we used a polyphasic approach for the identification of newly detected fungal strains, combining colony morphology, microscopy, MLST, and WGS. As a result, we describe three novel fungal strains and recommend the establishment of two new fungal genera and one novel species to accommodate the identified species. However, there were challenges in the interpretation and placement of novel fungi while comparing the taxonomy trees generated via MLST and WGS. The problems in setting up the concurrence are due to the absence of WGSs for the several strains that were considered in the generation of MLST. For example, the members of the family *Trichomeriaceae* were taken forward for MLST generation of *P. melaninifex*, which has defined the need to classify fungal species as novel (Figure 5). However, genomes of these members of *Trichomeriaceae* are either not available or not annotated yet to be considered forward. Therefore, the WGS-based phylogenomic tree was generated based on the available WGSs of members from other closely related families to confirm and validate the correct placement of *P. melaninifex* among related families. For the best placement and sensitivity of WGS-based phylogenomics in the taxonomy of novel fungal species, the WGSs of upcoming novel organisms being defined should be made available in the public domain. Thus, we recommend that every new fungal species being described as novel should have their WGS publicly available in order to gain more power and sensitivity for characterizing novel fungal organisms.

*Aaosphaeria pasadenensis: A. pasadenensis* is the new species of the genus *Aaosphaeria* (Figure 2). MLST tree aligns the new subclade near the genera *Roussoella* and *Neoroussoella,* which are characterized by a prominent sexual stage and the presence of septate ascospores. *A. pasadenensis* is distinguished from both closely related genera by the absence of a recognizable sexual stage. Asexual morphs (conidiomata) are observed like that of *N. bambusae* but the origin, shape, and size of conidiophores are unique in *A. pasadenensis* [80]. The conidiophores in *A. pasadenensis* arise from hyphal walls and are elongated as in *N. bambusae*, but are aseptate, thin, smooth-walled, and unbranched. These features warrant the recommendation of a novel species. The SEM images show the presence of conidiomata in strain *A. pasadenensis*, which resemble those observed in members of the genus *Liberomyces* [81]. Colony features such as the presence of unique conidiomata and morphology of conidiophores supported by SEM images are not reported in other closely related species. To the best of literature, the colony morphology of *A. pasadenensis* is dissimilar to *A. salicis* CBS 368.94 and *A. arxii* CBS 175.79, since unique multi-form anamorphs (distinguished conidiophore formations and conidia) are observed in *A. pasadenensis*. The six strains clustering under the new subclade in the MLST analysis are *A. arxii* CBS 175.79, *Roussoella ludipueritiae* CBS 170.96, and *A. salicis* strains (NRRL 62788, CBS 368.94, UTHSCDI16_220, and UTHSCDI16_356). Among these six strains, WGS was available only for *A. arxii* CBS 175.79. The WGS-based phylogeny constructed with representatives of various families showed that *A. pasadenensis* is different phylogenetically from *A. arxii* CBS 175.79 (Figure 3). Thus, based on distinct morphology, microscopic features, and phylogeny, the *A. pasadenensis* is recommended as the new species of the genus *Aaosphaeria*.

*Pasadenomyces melaninifex: P. melaninifex* is closely related to members of the genus *Lithohypha* (*Chaetothyriales, Trichomeriaceae* – Figure 5), but distinguished by the formation of sub-colonies on the existing base colony. The young arthroconidia seem more regular in shape (square to rectangular) on DIC images (Figure 4D) and somewhat similar to the vegetative hyphae of *Incumbomyces delicatus* [82]. Hyphal anastomosis is also observed, similar to that reported in *I. lentus* [82]. Thus, microscopic examination reveals more similarity to the members of the genus *Incumbomyces,* but with differences such as the presence of dumbbell-shaped mature hyphae which were not reported in most of the phylogenetically close related genera (*Lithohypha, Incumbomyces, Bradymyces,* and *Neostrelitziana*) [83]. These dumbbell-shaped mature hyphae, which are slightly swollen at both ends and have pear-shaped conidia formation at the apex of hyphae, are unique and help to classify *P. melaninifex* as a representative of the new genus. Interestingly, the dumbbell-shaped cells appear to emerge from a conidiogenous node (Figure 4K). According to the phylogenomic analysis of the WGS, *P. melaninifex* is a representative of *Trichomeriaceae* and should be placed in a more basal position of the tree, next to *Coccodiniaceae* and *Cyphellophoraceae* (Figure 6).

*Floridaphiala radiotolerans: F. radiotolerans* is closely related to *Kirschsteiniothelia aethiops* 109.53 of the order *Kirschsteiniotheliales, Verruconis gallopava* CBS 437.64 of the order *Venturiales,* members of the order *Trypetheliales* (*Astrothelium cinnamomeum* DUKE 0000007, *Trypethelium nitidiusculum* AFTOL 2099, and *Laurera megasperma* AFTOL 2094), *Lichenothelia convexa* LMXX0061 and *L. calcarean* L1324 of *Lichenotheliales,* and two species of the genus *Saxomyces* (*Saxomyces penninicus* CCFEE 5495 and *S. alpinus* CCFEE 5495). Microscopically, the chlamydoconidium-like cells of *F. radiotolerans* match most closely to the multiform and multicellular conidia of *K. rostrata* and *K. fluminicola*, observed at terminal conidiophores, whereas the chlamydoconidium-like cells in *F. radiotolerans* are mostly observed at intercalary positions [84]. In addition, the chlamydoconidium-like cells observed in *F. radiotolerans* contain endoconidium-like structures inside. Together, all these features distinguished the *F. radiotolerans* from members of the order *Kirschsteiniotheliales.* Less is known about the structural features of *V. gallopava,* which has been reported as the cause of pheohyphomycosis and even fungemia despite antifungal therapy [84]. The members of the order *Trypetheliales* are distinguished by the presence of varied forms of ascomata and multiseptate giant ascospores [85,86,87,88]. Members of order *Lichenotheliales* have two to four septate ascospores [89]. The genus *Lichenothelia* is characterized by fertile stromata and pycnidia which can grow on and within exposed rocks, optionally associating with algae, with some species also being lichenicolous [90]. The genus *Saxomyces* includes rock-inhabiting fungi and is characterized by a meristematic growth, a scarcely differentiated morphology with highly melanized thick-walled toruloid hyphae. Nonetheless, a few peculiar characteristics were also observed, such as convoluted hyphal tips and the production of spherical propagules in *Saxomyces* [91]. The *F. radiotolerans* is distinguished from most of these closely related orders due to the absence of a recognizable sexual stage and ascospores. However, chlamydoconidium-like cells of *F. radiotolerans* appear similar to ascospores in some phylogenetically close relatives described above. The *F. radiotolerans* is mapped between *Microthyriales* and *Trypetheliales* and potentially indicates that this species might constitute a novel class/family within *Dothideomycetes;* however, we have used a more conservative nomenclature and defined this as *incertae sedis* (Figure 3). 

In the *A. pasadenensis* genome, we predicted the presence of BGCs with 100% match to known clusters related to the synthesis of a siderophore and the virulence promoter dimethyl coprogen [61], the anti-tumor compound clavaric acid [62], and melanin. However, similar sets of gene clusters are also present in *A. arxii* CBS175.79 (Supplemental Table S4). In the *P. melaninifex* genome, 11 BGCs were identified including 5 NRPSs, 3 T1PKS, 2 terpenes, and one combination of NRPS and T1PKS. Strong (100%) matches to known BGCs include those for melanin, the radical-scavenging compound pyranonigrin E [68], and the industrial pigment monascorubrin [69]. In the *F. radiotolerans* genome, 12 BGCs were identified, including 3 NRPS/NRPS-like clusters, 6 T1PKS, and 3 terpene clusters. The only predicted gene cluster with a 100% match to known gene clusters is that of melanin, which is common in all three organisms [92]. The presence of melanin-related gene clusters in our strains may be the reason for their resistance to 2000 to 3000 J/m^2^ of UV-C dose but more research is warranted to unearth the molecular mechanisms. Detection of UV-resistant microbes in SAF is a concern for Planetary Protection that needs remedial plans such as antifungal cleaning reagents to contain forward contamination for future NASA life-detection missions.

## 5. Conclusions

Three strains isolated from NASA cleanrooms were described using conventional morphology and microscopy techniques and state-of-the-art molecular methods. *A. pasadenensis,* isolated from the JPL-SAF and six other isolates, not well-described representatives of the genera *Aosphaeria, Arthopyrenia,* and *Roussoella* or misidentified for their phylogeny were placed together in this group. *P. melaninifex*, another JPL-SAF isolate, revealed a new phylogenetic clade including members of the family *Trichomeriaceae.* Even though *P. melaninifex* was morphologically and phylogenetically distant from the fungal species belonging to the genera *Lithohypha* and *Strelitziana,* they were its closest relatives among *Trichomeriaceae* members. Finally, *F. radiotolerans,* the KSC-PHSF isolate was phylogenetically related to members of the family *Kirschsteiniotheliaceae*; however, no fungal genera were found closely related. Furthermore, the *F. radiotolerans* exhibited a distant phylogenetic relationship to the members of the order *Venturiales* and *Trypetheliales.* Predicted BGCs such as the anti-tumor compound clavaric acid in *A. pasadenensis*, as well as the radical-scavenging compound pyranonigrin E and the industrial pigment monascorubrin in *P. melaninifex*, warrant future studies to validate the genomic prediction by metabolome analysis. In addition, melanin production was predicted in all three novel strains. This functional property should be explored further along with other traits that may enable these fungi to survive and proliferate under extreme environmental conditions of interest to future NASA missions.

## Figures and Tables

**Table 1:** Genome features and other genomic annotations of three novel strains.

Novel Fungal Strains	*Aaosphaeria pasadenensis* (FJI-L9-BK-P1)	*Pasadenomyces melaninifex* (FJII-L3-CM-DR1)	*Floridaphiala radiotolerans* (FKI-L1-BK-DR1)
Number of contigs	122	972	109
Length	38,798,968	27,602,062	24,887,818
N50	943,274	498,971	518,619
L50	14	17	17
N90	314,675	48,404	169,985
L90	40	74	49
GC content (%)	49.73	48.13	52.18
Genes	12,248	10,961	8582
mRNA	12,141	10,856	8505
tRNA	107	105	77
Average gene length	1605.34	1536.55	1708.37
Total exons	33,005	20,143	22,516
Average exon length	467.7	545.7	512.5
Average protein length	492.79	488.12	526.9
GO terms	2295	2,107	2099
Interproscan	3140	2968	2911
Eggnog	11,235	9309	7651
Pfam	8359	7070	5866
CAZYme	588	297	266
MEROPS	395	346	257
BUSCO	3682	2928	3412
Secretion	1204	604	578

## Data Availability

The draft genomes JAKLMB000000000, JAKLMI000000000, and JAKLMZ000000000 and raw data (SRR18739370, SRR18739410, and SRR18739394), have been deposited in GenBank under the BioProject accession number PRJNA800051.

## Supplementary Materials

The following supporting information can be downloaded at: https://www.mdpi.com/article/10.3390/jof9010031/s1, Table S1: Marker gene table for *Aaosphaeria pasadenensis* FJI-L9-BK-P1; Table S2: Marker gene table for *Pasadenomyces melaninifex* FJII-L3-CM-DR1; Table S3: Marker Gene table for *Floridaphiala radiotolerans* FKI-L1-BK-DR1; Table S4: Genomic annotations related to BGCs; Table S5: Proteomics data utilized in developing WGS-based phylogenomic tree.
