# Supplementary material for: Description and Genome Characterization of Three Novel Fungal Strains Isolated from Mars 2020 Mission-Associated Spacecraft Assembly Facility Surfaces—Recommendations for Two New Genera and One Species
Source: J Fungi (Basel). 2022 Dec 23;9(1):31. doi: 10.3390/jof9010031 (PMC9864340; doi:10.3390/jof9010031)
Supplement: Supplementary file 1 [file jof-09-00031-s001.zip › 10. Table S1 Maker genes.pdf]

Supplemental Table S1: Marker gene table for *Aaosphaeria pasadenensis* FJI-L9-BK-P1; N/A- not available

**Genus**	**Species**	**Strain**	***LSU***	***ITS***	***TEF* 1**	***RPB* 2**
Arthopyrenia	*sp.*	UTHSC DI16–362	LN907505	LT796905	LT797145	LT797065
Arthopyrenia	*sp.*	UTHSC DI16–334	LN907477	LT796887	LT797127	LT797047
Neoroussoella	*alishanense*	FU31016	MK503822	MK503816	MK336181	MN037756
Neoroussoella	*bambusae*	MFLUCC 11–0124	KJ474839	KJ474827	KJ474848	KJ474856
Neoroussoella	*sp.*	MFLUCC 15–0098A	MH260309	MH275075	N/A	N/A
Neoroussoella	*heveae*	MFLUCC 17–1983	MH590689	MH590693	N/A	N/A
Neoroussoella	*lenispora*	GZCC 16-0020	KX791431	N/A	N/A	N/A
Neoroussoella	*leucaenae*	C356	MK347984	MK347767	MK360067	MK434876
Neoroussoella	*solani*	CPC 26331	KX228312	KX228261	N/A	N/A
Roussoella	*mukdahanensis*	HKAS 101766	MH453485	MH453489	MH453478	MH453482
Pararoussoella	*rosarum*	MFLUCC 17–0796	MG829048	MG828939.	MG829224	N/A
Parathyridaria	*ramulicola*	CBS 141479	KX650565	KX650565	KX650536	KX650584
Parathyridaria	*robiniae*	MFLUCC 14–1119	KY511141	KY511142	KY549682	N/A
Roussoella	*euonymi*	CBS 143426	MH107961	MH107915	N/A	MH108007
Pseudoneoconiothyrium	*rosae*	MFLU 18-0117	NG_059868	NR_157523	N/A	N/A
Pseudoroussoella	*chromolaenae*	MFLUCC 17–1492	MT214439	MT214345	MT235769	N/A
Pseudoroussoella	*elaeicola*	MFLUCC 17–1483	MT214442	MT214348	MT235772	MT235808
Pseudoroussoella	*elaeicola*	MFLUCC 15–0276b	MH742327	MH742330	N/A	N/A
Pseudoroussoella	*elaeicola*	MFLUCC 15–0276a	MH742326	MH742329	N/A	N/A
Roussoella	*angusta*	MFLUCC 15–0186	KT281979	N/A	N/A	N/A
Roussoella	*arundinacea*	CPC 35554	MT223928	MT223838	N/A	N/A
Roussoella	*chiangraina*	MFLUCC 10–0556	KJ474840	KJ474828	KJ474849	KJ474857
Roussoella	*doimaesalongensis*	MFLUCC 14–0584	KY000659	KY026584	KY651249	KY678394
Roussoella	*guttulata*	MFLUCC 20–0102	MT734818	MT734821	MW022188	MW022187
Roussoella	*hysterioides*	CBS 546.94	KF443381	KF443405	KF443399	KF443392
Roussoella	*intermedia*	NBRC 106245	AB524624	KJ474831	N/A	N/A
Roussoella	*japanensis*	MAFF 239636	AB524621	KJ474829	AB539114	AB539101
Roussoella	*kunmingensis*	HKAS 101773	MH453487	MH453491	MH453480	MH453484
Roussoella	*magnatum*	MFLUCC 15–0185	KT281980	N/A	N/A	N/A
Roussoella	*margidorensis*	MUT 5329	MN556322	KU314944	MN605897	MN605917
Roussoella	*mediterranea*	MUT 5369	MN556324	KU314947	MN605899	MN605919
Roussoella	*neopustulans*	MFLUCC 11–0609	KJ474841	KJ474833	KJ474850	N/A
Roussoella	*neopustulans*	MFLUCC 12–0003	KU863119	KU940130	N/A	N/A

Roussoella	*nitidula*	MFLUCC 11–0182	KJ474843	KJ474835	KJ474852	KJ474859
Roussoella	*nitidula*	MFLUCC 11–0634	KJ474842	KJ474834	KJ474851	KJ474858
Roussoella	*padinae*	MUT 5365	MN556327	KU158170	MN605902	MN605922
Roussoella	*padinae*	MUT5341	MN556325	KU158153	MN605900	MN605920
Roussoella	*pseudohysterioides*	MFLUCC 13–0852	KU863120	KU940131	KU940198	N/A
Roussoella	*pseudohysterioides*	HKAS 101758	MH453486	MH453490	MH453479	MH453483
Roussoella	*pustulans*	MAFF 239637	AB524623	KJ474830	AB539116	AB539103
Roussoella	*scabrispora*	MFLUCC 11–0624	KJ474844	KJ474836	KJ474853	KJ474860
Roussoella	*scabrispora*	MFLUCC 14–0582	KY000660	KY026583	N/A	N/A
Roussoella	*scabrispora*	RSC	KX650566	KX650566	KX650537	N/A
Roussoella	*siamensis*	MFLUCC 11–0149	KJ474845	KJ474837	KJ474854	KJ474861
Roussoella	*thailandica*	MFLUCC 11–0621	KJ474846	KJ474838	N/A	N/A
Roussoella	*tuberculata*	MFLUCC 13–0854	KU863121	KU940132	KU940199	N/A
Roussoella	*verrucispora*	CBS 125434	AB524622	KJ474832	AB539115	AB539102
Roussoella	*sp.*	HKAS-101762-HKAS	MH453488	MH453492	MH453481	N/A
Roussoellopsis	*macrospora*	MFLUCC 12–0005	KJ474847	N/A	KJ474855	KJ474862
Roussoellopsis	*sp.*	KT 1710	AB524626	N/A	N/A	N/A
Roussoellopsis	*tosaensis*	KT 1659	AB524625	N/A	AB539117	AB539104
Setoarthopyrenia	*chromolaenae*	MFLUCC 17–1444	MT214438	MT214344	MT235768	MT235805
Roussoella	*acaciae*	CBS 138873	KP004497	KP004469	N/A	N/A
Thyridaria	*broussonetiae*	CBS 141481	KX650568	NR_147658	KX650539	KX650586
Torula	*herbarum*	CBS 111855	KF443386	KF443409	KF443403	KF443396
Torula	*hollandica*	CBS 220.69	KF443384	KF443406	KF443401	KF443393
Xenoroussoella	*triseptata*	MFLUCC 17–1438	MT214437	MT214343	MT235767	MT235804
Ohleria	*modesta*	MGC	KX650562	KX650562	KX650533	KX650582
Ohleria	*modesta*	CBS 141480	KX650563	KX650563	KX650534	KX650583
Dendryphion	*europaeum*	CPC 22943	KJ869203	KJ869146	N/A	N/A
Nigrograna	*fuscidula*	CBS 141476	KX650547	KX650547	KX650522	KX650576
Nigrograna	*mackinnonii*	CBS 110022	GQ387614	KF015653	KF407985	KF015704
Nigrograna	*mycophila*	CBS 141478	KX650553	KX650553	KX650526	N/A
Nigrograna	*norvegica*	CBS 141485	KX650556	KX650556	N/A	KX650578
Nigrograna	*obliqua*	CBS 141475	KX650558	KX650558	KX650530	KX650579
Arthopyrenia	*salicis*	CBS 368.94	AY538339	KF443410	KF443404	KF443397
Arthopyrenia	*salicis*	NRRL62788	N/A	KM030296	N/A	N/A
Roussoella	*intermedia*	CBS 170.96	KF443382	KF443407	KF443398	KF443394

Aaosphaeria	*arxii*	CBS 175.79	OU989224	NR_169685	N/A	N/A
Arthopyrenia	*salicis*	UTHSC:DI16-*356*	LN907499	LT796900	LT797140	LT797060
Arthopyrenia	*salicis*	UTHSCDI16_220	LN907363	LT796841	LT797081	LT797001
