# Supplementary material for: Description and Genome Characterization of Three Novel Fungal Strains Isolated from Mars 2020 Mission-Associated Spacecraft Assembly Facility Surfaces—Recommendations for Two New Genera and One Species
Source: J Fungi (Basel). 2022 Dec 23;9(1):31. doi: 10.3390/jof9010031 (PMC9864340; doi:10.3390/jof9010031)
Supplement: Supplementary file 1 [file jof-09-00031-s001.zip › 11. Table S2 Marker genes.pdf]

Supplemental Table S2: Marker gene table for *Pasadenomyces melaninifex* FJII-L3-CM-DR1; N/A- not available

**Species name**	**Strain**	***ITS***	***LSU***	***TEF* 1**	***RPB* 1**	***TUB***
Anthracinomyces petraeus	CGMCC 3.17315	KP174843.1	KP174924.1	KP174998.1		KP226555.1
Anthracinomyces ramosus	CGMCC 3.16367	KP174846.1	KP174922.1	KP175001.1	KP226535.1	KP226556.1
Knufia perforans	CBS 885.95 (T)	MH862564.1	NG042586.1	N/A	N/A	N/A
Knufia petricola	CBS 726.95 (T)	MH862556.1	NG042775.1	N/A	N/A	N/A
Knufia vaticanii	CCFEE 5939 (T)	KP791780.1	KR781068.1	N/A	N/A	N/A
Knufia marmoricola	CCFEE 6201	KP791790.1	KR781077.1	N/A	N/A	N/A
Knufia karalitana	CCFEE 5921	KP791784.1	KR781072.1	N/A	N/A	N/A
Knufia epidermidis	CBS 120353 (T)	NR_111330.1	NG042475.1	EU884530.1	N/A	N/A
Knufia cryptophialidica	DAOM 216555 (T)	JN040501.1	JN040500.1	N/A	N/A	N/A
Knufia mediterranea	CBS 139721 (T)	KP791794.1	KR781081.1	N/A	N/A	N/A
Arthrocladium tropicale	CBS 134926 (T)	KX822543.1	NG057119.1	KT806378.1	KX822573.1	KX822406.1
Arthrocladium tardum	CBS 127021 (T)	KT337440.1	NG057089.1	KT806376.1	N/A	N/A
Arthrocladium caudatum	CBS 457.67 (T)	MH859032.1	NG_057084	KT806374.1	LT558711.1	LT558710.1
Arthrocladium fulminans	CBS 136243 (T)	KT337439.1	NG_057084	KT806375.1	N/A	N/A
Chaetothyriales sp.	CBS 128958	KX822541	KX822541	KX822464.1	N/A	N/A
Chaetothyriales sp.	CBS 129049	KX822531	KX822531	KX822458.1	N/A	N/A
Chaetothyriales sp.	CBS 129047	KX822533.1	KX822533	N/A	N/A	N/A
Exophiala placitae	CBS 121716 (T)	MH863143.1	MH874694.1	N/A	N/A	N/A
Cladophialophora eucalypti	CBS 145551 (T)	MK876380.1	MK876419.1	N/A	N/A	N/A
Cladophialophora pucciniophila	KUS F23645	JF263533.1	JF263534.1	N/A	N/A	N/A
Cladophialophora proteae	CBS 111667 (T)	EU035411.1	EU035411.1	N/A	KJ636044.1	N/A
Strelitziana albiziae	CBS 126497 (T)	MH864122.1	HQ599585.1	N/A	N/A	N/A
Strelitziana eucalypti	CBS 128214	HQ599596.1	HQ599597.1	N/A	N/A	N/A
Strelitziana australiensis	CBS 124778 (T)	GQ303295.1	GQ303326.2	GU384362.1	N/A	N/A
Strelitziana cliviae	CPC 19822 (T)	KC005772.1	NG042750.1	N/A	N/A	N/A
Bradymyces alpinus	CCFEE 5493 (T)	HG793052.1	GU250396.1	N/A	LT558712.1	LN589970.1
Bradymyces graniticola	F6A	KX179910.1	KX179912.1	N/A	N/A	N/A
Bradymyces oncorhynchi	CCF 4369 (T)	NR_132843.1	NG058643.1	HG426061.1	LT558713.1	HG426060.1
Trichomerium foliicola	MFLUCC 10-0078 (T	JX313655.1	JX313661.1	N/A	N/A	N/A
Trichomerium gloeosporum	MFLUCC 10-0087 (T	JX313656.1	JX313662.1	N/A	N/A	N/A
Trichomerium dioscoreae	CBS 138870 (T)	NR_137946.1	NG058126.1	N/A	N/A	N/A
Trichomerium deniquelatum	MFLUCC 10-0884 (T	JX313654.1	JX313660.1	N/A	N/A	N/A
Trichomerium eucalypti	CBS 143443 (T)	NR_156672.1	NG058525.1	N/A	N/A	N/A
Knufia peltigerae	CGMCC:3.17283	KP174864.1	KP174935.1	KP175006.1	KP226513.1	KP226562.1
Knufia tsunedae	FMR 10621 (T)	NR_132842.1	HG003672.1	N/A	N/A	N/A
Metulocladosporiella musicola	CBS 110960 (T)	MH862870.1	DQ008153.1	MG934484.1	N/A	N/A
Brycekendrickomyces acaciae	CBS 124104 (T)	NR_132828.1	NG_058633.1	N/A	N/A	N/A
Exophiala encephalarti	CBS 128210	HQ599588.1	HQ599589.1	N/A	N/A	N/A
Ceramothyrium melastoma	CPC 19837 (T)	NR_111822.1	NG_042749.1	N/A	N/A	N/A
Neostrelitziana acaciigena	CBS 139903(T)	NR_137987.1	NG_058165.1	N/A	N/A	N/A
Strelitziana africana	CBS 120037	DQ885895.1	DQ885895.1	N/A	N/A	N/A
Lithohypha aloicola	CPC 35996(T)	NR_166313.1	MN567611.1	MN556829.1	N/A	MN556837.1
Bacillicladium clematidis	CBS:145035	NR_163355.1	MK442512.1		N/A	MK442726.1
Lithohypha catenulata	CGMCC:3.14008	KP174841.1	KP174841.1	KP174996.1	KP226519.1	KP226543.1
Lithohypha guttulata	CCFEE 5907	KP791773.1	KR781061.1	N/A	N/A	N/A
Knufia calcarecola	CGMCC:3.17218	N/A	NG_074883.1	KP175008.1	KP226506.1	N/A
Knufia separata	CGMCC 3.17337	KP174856.1	KP174932.1	KP175003.1	KP226516.1	KP226564.1
Knufia walvisbayicola	CBS 146989	NR_173046.1	NG_076737.1	N/A	N/A	N/A
Strelitziana malaysiana	CBS:139902	NR_137988.1	NG_058166.1	N/A	N/A	N/A
Strelitziana cliviae	CPC 19822	NR_111823	NG_042750.1	N/A	N/A	N/A
Strelitziana sarbhoyi	NFCCI 4772	NR_172975.1	NG_076696.1	N/A	N/A	N/A
Strelitziana syzygii	CPC 26591	NR_145400.1	NG_059619.1	KT950881.1	N/A	KT950884.1
Metulocladosporiella musae	CBS 161.74	AY186199.1	DQ008161.1	MG934478.1	N/A	N/A
Trichomerium bambusae	MFLU 16-2286	NR_155921.1	N/A	N/A	N/A	N/A
Trichomerium camporesii	MFLU 19-2251	NR_169997.1	MN644511.1	N/A	N/A	N/A
Knufia sp. FH-2012	BJ01A29	KF589944.1	KF589944.1	N/A	N/A	N/A
Knufia perfecta	IRAN 2553C	MF062036.1	MF062037.1	N/A	N/A	N/A
Knufia hypolithi	CBS 146991	NR_173045.1	NG_076736.1	N/A	N/A	N/A
Knufia endospora	UAMH 10396	NR_111634.1	JN040510.1	N/A	N/A	N/A
Trichomerium cicatricatum	CGMCC 3.17307	N/A	N/A	KP175025.1	KP226531.1	KP226540.1
Trichomerium flexuosum	CGMCC 3.17988	NR_172253.1	NG_075211.1		KX348490.1	KX348496.1
Trichomerium lapideum	CGMCC 3.17311	NR_172231.1	KP174850.1	KP175023.1	KP226528.1	KP226539.1

Trichomerium leigongense	CGMCC 3.17983	NR_172254.1	KX348470.1	KX348493.1	KX348491.1	KX348497.1
Trichomerium syzygii	CPC 37184	NR_170066.1	NG_074428.1	N/A	N/A	N/A
Fonsecaea pedrosoi	CBS 271.37	Extracted	Extracted	Extracted	Extracted	Extracted
Incumbomyces lentus	CBS 128958	NR_176739.1	MT757423.1	KX822464.1	N/A	N/A
Incumbomyces delicatus	CBS 129047	NR_176738.1	MT757422.1	N/A	N/A	N/A
