# Supplementary material for: Description and Genome Characterization of Three Novel Fungal Strains Isolated from Mars 2020 Mission-Associated Spacecraft Assembly Facility Surfaces—Recommendations for Two New Genera and One Species
Source: J Fungi (Basel). 2022 Dec 23;9(1):31. doi: 10.3390/jof9010031 (PMC9864340; doi:10.3390/jof9010031)
Supplement: Supplementary file 1 [file jof-09-00031-s001.zip › 12. Table S3 Marker genes.pdf]

Supplemental Table S3: Marker Gene table for *Floridaphiala radiotolerans* FKI-L1-BK-DR1**;** N/A- not available

Genus	Species	Strain	*LSU*	*ITS*	*TEF1*	*RPB2*	*TUB*
Elasticomyces	*elasticus*	CCFEE 5490	KF309992	KF309951	N/ A	KF310047	KF546746
Friedmanniomyces	*endolithicus*	CCFEE 524	GU250364	JN88554	N/ A	KF310054	KF546732
Recurvomyces	*mirabilis*	CCFEE 5475	KC315876	KF309962	N/ A	KF310060	N/ A
Mycosphaerella	*fijiensis*	AFTOL 2021	DQ678098	N/ A	N/ A	DQ677993	N/ A
Capnodium	*salicinum*	CBS 131.34	MH866941	MH855469	DQ677889	KT216553	N/ A
Capnodium	*coffeae*	CBS 147.52	MH868489	MH856967	DQ471089	KF902196	N/ A
Phaeotheca	*fissurella*	CBS 520.89	MH873872	MH862184	MN829428	MN829342	N/ A
Stylodothis	*puccinioides*	CBS 193.58	MH869286	MH857753	DQ677886		N/ A
Dothidea	*insculpta*	CBS 189.58	MH869284	AF027764	DQ471081	DQ247792	N/ A
Sydowia	*polyspora*	CBS 116.29	MH866487	MH855019	DQ677899	GU357791	N/ A
Elsinoe	*centrolobi*	CBS 222.50	NG_069000	MH856595	DQ677934	KX887089	N/ A
Myriangium	*duriaei*	CBS 260.36	OM238138	MH855793	DQ677900	KT216528	N/ A
Saxomyces	*alpinus*	CCFEE 5466	GU250392	N/ A	N/ A	N/ A	N/ A
Saxomyces	*penninicus*	CCFEE 5495	KC315875	N/ A	N/ A	N/ A	N/ A
Lichenothelia	*convexa*	LMCC0061	KR045750	Mycocosm	Mycocosm	N/ A	Mycocosm
Lichenothelia	*calcarea*	L1324	KC015062	N/ A	N/ A	N/ A	N/ A
Laurera	*megasperma*	AFTOL 2094	FJ267702	N/ A	N/ A		N/ A
Trypethelium	*nitidiusculum*	AFTOL 2099	FJ267701	N/ A	GU327732	GU561856	N/ A
Astrothelium	*cinnamomeum*	DUKE 0000007	AY584652	AY584652	DQ782896	AY584690	N/ A
Verruconis	*gallopava*	CBS 437.64	NG_058016	HQ667553	KF155968	AB569127	KF156203
Kirschsteiniothelia	*aethiops*	CBS 109.53	MH869065	MH857990	DQ471084	DQ470914	
Leptosphaeria	*maculans*	CBS 275.63	MH869888	MH858282	MW735661	KT389669	KT389841
Pleospora	*herbarum*	CBS 191.86	JX681120	MH861935	KC584731	KC584471	AY749032
Westerdykella	*cylindrical*	CBS 454.72	NG_027595	N/ A	N/ A	N/ A	N/ A
Hysterobrevium	*smilacis*	CBS 114601	FJ161174	N/ A	FJ161091	GU357806	N/ A
Gloniopsis	*praelonga*	CBS 112415	FJ161173	N/ A	FJ161090	FJ161113	N/ A
Anisomeridium	*polypori*	AFTOL 101	DQ782906	DQ782838	DQ782894	DQ782864	N/ A
Lophium	*mytilinum*	CBS 269.34	DQ678081	EF596817	DQ677926	GU456342	Mycocosm
Mytilinidion	*resinicola*	CBS 304.34	MH867038	MH855535	FJ161101	FJ161120	N/ A
Diplodia	*corticola*	CBS 112549	KF766323	KF766156	KF766398	KX46395	DQ458853
Neofusicoccum	*ribis*	CBS 115475	DQ678053	KF766205	DQ677893	GU357789	
Dothiorella	*ulmicola*	CPC 24416	KR611899	KR611881	KR611910	N/ A	KR611909
Botryosphaeria	*dothidea*	CBS 115476	NG_027577	KF766151	DQ767637	GU357802	N/ A
Abrothallus	*cladoniae*	AB53	KF816228	KF816173	KF816181	KF816200	N/ A
Abrothallus	*parmotrematis*	AB1	KF816231	KF816176	KF816185.1	KF816204	
Kellermania	*confusa*	CBS 131723	KF766344	NR_165953	KF766405	N/ A	KF766134
Macrophomina	*phaseolina*	CBS 227.33	DQ377906	KF951627	KF952000	N/ A	KF531806
Saccharata	*capensis*	CBS 122693	N/ A	KF766224	N/ A	N/ A	KX465073
Saccharata	*leucospermi*	CBS 122694	NR_168142	EU552129	EU552094	KX464073	
Rupestriomyces	*sinensis*	CGMCC 3.17067	KF680788	KF513525	N/ A	KJ733740	KJ733715
Rupestriomyces	*torulosus*	CGMCC 3.17054	KF680792	KF513528	N/ A		KJ733710
Rupestriomyces	*ampulliformis*	CGMCC 3.17063	KF680798	KF513537	N/ A	KJ733739	KJ733713
Spissiomyces	*aggregatus*	CGMCC 3.17072	NG_059959	KF513518	N/ A	KJ733736	KJ733706
Spissiomyces	*ramosus*	CGMCC 3.17077	KF680783	KF513515		KJ733734	KJ733700
Helicomyces	*roseus*	CBS 283.51	DQ678083	AY916464	DQ677928	DQ677981	N/ A
Tubeufia	*paludosa*	CBS 120503	GU301877	N/ A	GU349024	GU357754	N/ A
Tubeufia	*amazonensis*	ATCC 42524	AY787938	AY916458	N/ A	N/ A	N/ A
Tubeufia	*cerea*	CBS 254.75	DQ470982	N/ A	DQ471105	DQ470934	N/ A
Coniosporium	*uncinatum*	CBS 100219	NG_058806	NR_145343	N/ A	GU250957	N/ A
Coniosporium	*apollinis*	CBS 109860	GU250899	N/ A	Mycocosm	GU250953	Mycocosm

Schismatomma	*decolorans*	AFTOL 307	AY548815	N/ A	DQ883725	DQ883715	N/ A
Hysteropatella	*clavispora*	CBS 247.34	AY541483	N/ A	DQ677901	DQ677955	N/ A
Cryomyces	*minteri*	CCFEE 5187	NG_058847	Mycocosm	Mycocosm	N/ A	N/ A
Cryomyces	*antarcticus*	CCFEE 534	Mycocosm	Mycocosm	Mycocosm	N/ A	Mycocosm
Cryomyces	*montanus*	CCFEE 5476	NG_073589	NR_171791	N/ A	N/ A	N/ A
Cryomyces	*funiculosus*	CCFEE 5554	N/ A	NR_171790	N/ A	N/ A	N/ A
Haudseptoria	*typhae*	CPC 38203	MW883815	MW883421	N/ A	N/ A	MW890131
Asterodiscus	*tamaricis*	L114	KU234101	KU234101	KU234133	KU234116	KU234135
Jahnula	*sangamonensis*	A482-1B	EF175662	JN942350	N/ A	N/ A	N/ A
Jahnula	*aquatica*	R68-1	EF175655	JN942354	N/ A	N/ A	N/ A
Aliquandostipite	*khaoyaiensis*	ISAN100	MT860428	MT864350	MT873577	MT873578	N/ A
