# Supplementary material for: Description and Genome Characterization of Three Novel Fungal Strains Isolated from Mars 2020 Mission-Associated Spacecraft Assembly Facility Surfaces—Recommendations for Two New Genera and One Species
Source: J Fungi (Basel). 2022 Dec 23;9(1):31. doi: 10.3390/jof9010031 (PMC9864340; doi:10.3390/jof9010031)
Supplement: Supplementary file 1 [file jof-09-00031-s001.zip › 13. Table S4 biosynthetic gene clusters.pdf]

| **Supplemental Table S4:** Genomic annotations related to BGCs | | | | | | | |
|---|---|---|---|---|---|---|---|
| **Strain** | **Region** | **Type** | **From** | **To** | **Most similar known cluster** | **Product** | **Similarity** |
| *Pasadenomyces melaninifex* | Region 3.1 | NRPS-like | 349990 | 393358 | -- | -- | -- |
| *Pasadenomyces melaninifex* | Region 11.1 | NRPS,T1PKS | 125481 | 177247 | pyranonigrin E | Polyketide | 100.00% |
| *Pasadenomyces melaninifex* | Region 17.1 | T1PKS | 89094 | 135684 | melanin | Polyketide | 100.00% |
| *Pasadenomyces melaninifex* | Region 18.1 | NRPS | 197626 | 240901 | -- | -- | -- |
| *Pasadenomyces melaninifex* | Region 31.1 | NRPS-like | 135644 | 179477 | -- | -- | -- |
| *Pasadenomyces melaninifex* | Region 53.1 | T1PKS | 1 | 39566 | -- | -- | -- |
| *Pasadenomyces melaninifex* | Region 57.1 | NRPS | 3352 | 49900 | -- | -- | -- |
| *Pasadenomyces melaninifex* | Region 58.1 | T1PKS | 24716 | 89331 | monascorubrin | Polyketide | 100.00% |
| *Pasadenomyces melaninifex* | Region 87.1 | terpene | 32507 | 53565 | squalestatin S1 | Terpene | 40.00% |
| *Pasadenomyces melaninifex* | Region 97.1 | terpene | 1386 | 25685 | -- | -- | -- |
| *Pasadenomyces melaninifex* | Region 196.1 | NRPS | 1 | 26175 | -- | -- | -- |
| *Aaosphaeria pasadenensis* | Region 1.1 | NRPS-like | 1725559 | 1770580 | -- | -- | -- |
| *Aaosphaeria pasadenensis* | Region 2.1 | NRPS | 680924 | 724851 | -- | -- | -- |
| *Aaosphaeria pasadenensis* | Region 2.2 | T1PKS | 973844 | 1020487 | -- | -- | -- |
| *Aaosphaeria pasadenensis* | Region 4.1 | T1PKS | 615603 | 663168 | -- | -- | -- |
| *Aaosphaeria pasadenensis* | Region 4.2 | terpene | 1104935 | 1126541 | squalestatin S1 | Terpene | 40.00% |
| *Aaosphaeria pasadenensis* | Region 5.1 | terpene | 1 | 17641 | -- | -- | -- |
| *Aaosphaeria pasadenensis* | Region 5.2 | NRPS | 70293 | 134531 | -- | -- | -- |
| *Aaosphaeria pasadenensis* | Region 6.1 | NRPS | 1250532 | 1295790 | dimethylcoprogen | NRP | 100.00% |
| *Aaosphaeria pasadenensis* | Region 6.2 | terpene | 1315017 | 1337407 | clavaric acid | Terpene | 100.00% |
| *Aaosphaeria pasadenensis* | Region 8.1 | terpene | 1200299 | 1221511 | -- | -- | -- |
| *Aaosphaeria pasadenensis* | Region 9.1 | indole | 1071707 | 1092388 | -- | -- | -- |
| *Aaosphaeria pasadenensis* | Region 11.1 | NRPS | 221640 | 264697 | -- | -- | -- |
| *Aaosphaeria pasadenensis* | Region 13.1 | NRPS-like | 686312 | 730178 | -- | -- | -- |
| *Aaosphaeria pasadenensis* | Region 14.1 | T1PKS | 156012 | 199095 | -- | -- | -- |
| *Aaosphaeria pasadenensis* | Region 14.2 | T1PKS | 658101 | 699665 | -- | -- | -- |
| *Aaosphaeria pasadenensis* | Region 14.3 | T1PKS | 749235 | 797425 | -- | -- | -- |
| *Aaosphaeria pasadenensis* | Region 15.1 | T1PKS | 3476 | 38300 | -- | -- | -- |
| *Aaosphaeria pasadenensis* | Region 15.2 | T1PKS,indole | 588536 | 639094 | secalonic acids | Polyketide | 37.00% |
| *Aaosphaeria pasadenensis* | Region 19.1 | T1PKS | 192751 | 241418 | -- | -- | -- |
| *Aaosphaeria pasadenensis* | Region 24.1 | NRPS | 109263 | 158391 | -- | -- | -- |
| *Aaosphaeria pasadenensis* | Region 27.1 | NRPS-like | 262686 | 307100 | -- | -- | -- |
| *Aaosphaeria pasadenensis* | Region 30.1 | T1PKS | 135821 | 182426 | melanin | Polyketide | 100.00% |
| *Aaosphaeria pasadenensis* | Region 31.1 | NRPS-like | 124917 | 169245 | -- | -- | -- |
| *Aaosphaeria pasadenensis* | Region 33.1 | terpene | 190431 | 211347 | -- | -- | -- |
| *Aaosphaeria pasadenensis* | Region 35.1 | T1PKS | 189828 | 232853 | -- | -- | -- |
| *Aaosphaeria pasadenensis* | Region 35.2 | T1PKS | 314011 | 360756 | -- | -- | -- |
| *Aaosphaeria pasadenensis* | Region 41.1 | NRPS-like | 77044 | 123777 | -- | -- | -- |
| *Aaosphaeria pasadenensis* | Region 44.1 | NRPS | 24513 | 80040 | -- | -- | -- |
| *Aaosphaeria pasadenensis* | Region 46.1 | T1PKS | 129843 | 194763 | chaetoviridin E | Polyketide | 16.00% |
| *Aaosphaeria pasadenensis* | Region 50.1 | indole,NRPS-like | 140456 | 191535 | -- | -- | -- |
| *Aaosphaeria arxii* | Region 5.1 | NRPS | 140386 | 186264 | dimethylcoprogen | NRP | 100.00% |
| *Aaosphaeria arxii* | Region 5.2 | terpene | 203205 | 225595 | clavaric acid | Terpene | 100.00% |
| *Aaosphaeria arxii* | Region 8.1 | NRPS | 52423 | 95570 | -- | -- | -- |
| *Aaosphaeria arxii* | Region 26.1 | terpene | 136652 | 158621 | squalestatin S1 | Terpene | 40.00% |
| *Aaosphaeria arxii* | Region 38.1 | T1PKS | 1 | 60011 | toviridin E / 11-epichaetomugil | Polyketide | 22.00% |
| *Aaosphaeria arxii* | Region 40.1 | NRPS | 38102 | 88208 | vancomycin | NRP | 8.00% |
| *Aaosphaeria arxii* | Region 45.1 | NRPS | 291459 | 354667 | -- | -- | -- |
| *Aaosphaeria arxii* | Region 50.1 | T1PKS | 1 | 24106 | -- | -- | -- |
| *Aaosphaeria arxii* | Region 53.1 | T1PKS,indole | 101374 | 151980 | secalonic acids | Polyketide | 37.00% |
| *Aaosphaeria arxii* | Region 54.1 | NRPS-like | 135055 | 175907 | -- | -- | -- |
| *Aaosphaeria arxii* | Region 60.1 | indole | 92569 | 113420 | -- | -- | -- |
| *Aaosphaeria arxii* | Region 60.2 | T1PKS | 1457427 | 1504026 | melanin | Polyketide | 100.00% |
| *Aaosphaeria arxii* | Region 63.1 | NRPS | 14427 | 59881 | -- | -- | -- |
| *Aaosphaeria arxii* | Region 68.1 | NRPS-like | 206722 | 250588 | -- | -- | -- |
| *Aaosphaeria arxii* | Region 75.1 | T1PKS | 51593 | 99056 | -- | -- | -- |
| *Aaosphaeria arxii* | Region 77.1 | T1PKS | 397785 | 439843 | -- | -- | -- |

| | | | | | | | |
|---|---|---|---|---|---|---|---|
| *Aaosphaeria arxii* | Region 77.2 | T1PKS | 491234 | 537886 | -- | -- | -- |
| *Aaosphaeria arxii* | Region 79.1 | T1PKS | 402924 | 450562 | -- | -- | -- |
| *Aaosphaeria arxii* | Region 83.1 | T1PKS | 322954 | 370951 | -- | -- | -- |
| *Aaosphaeria arxii* | Region 87.1 | T1PKS | 37166 | 85220 | -- | -- | -- |
| *Aaosphaeria arxii* | Region 93.1 | T1PKS | 56062 | 102432 | -- | -- | -- |
| *Aaosphaeria arxii* | Region 94.1 | NRPS | 31365 | 59040 | -- | -- | -- |
| *Aaosphaeria arxii* | Region 100.1 | T1PKS | 1 | 11992 | -- | -- | -- |
| *Aaosphaeria arxii* | Region 109.1 | NRPS-like | 120410 | 163741 | -- | -- | -- |
| *Aaosphaeria arxii* | Region 112.1 | T1PKS | 1 | 30025 | -- | -- | -- |
| *Aaosphaeria arxii* | Region 153.1 | terpene | 117985 | 138852 | -- | -- | -- |
| *Aaosphaeria arxii* | Region 153.2 | indole,NRPS-like | 459684 | 517023 | -- | -- | -- |
| *Aaosphaeria arxii* | Region 159.1 | NRPS | 142490 | 198017 | -- | -- | -- |
| *Floridaphiala radiotolerans* | Region 4.1 | T1PKS | 862238 | 908800 | melanin | Polyketide | 100.00% |
| *Floridaphiala radiotolerans* | Region 6.1 | T1PKS | 665318 | 715085 | -- | -- | -- |
| *Floridaphiala radiotolerans* | Region 7.1 | terpene | 325151 | 346565 | -- | -- | -- |
| *Floridaphiala radiotolerans* | Region 8.1 | terpene | 185241 | 206665 | -- | -- | -- |
| *Floridaphiala radiotolerans* | Region 13.1 | terpene | 23781 | 48452 | -- | -- | -- |
| *Floridaphiala radiotolerans* | Region 14.1 | NRPS | 168805 | 224504 | -- | -- | -- |
| *Floridaphiala radiotolerans* | Region 15.1 | NRPS-like | 29789 | 73426 | -- | -- | -- |
| *Floridaphiala radiotolerans* | Region 19.1 | T1PKS | 221337 | 269747 | -- | -- | -- |
| *Floridaphiala radiotolerans* | Region 36.1 | T1PKS | 1 | 46989 | -- | -- | -- |
| *Floridaphiala radiotolerans* | Region 36.2 | NRPS-like | 124957 | 168130 | -- | -- | -- |
| *Floridaphiala radiotolerans* | Region 44.1 | T1PKS | 147713 | 182118 | -- | -- | -- |
| *Floridaphiala radiotolerans* | Region 52.1 | T1PKS | 33605 | 83421 | -- | -- | -- |
