# Supplementary material for: Description and Genome Characterization of Three Novel Fungal Strains Isolated from Mars 2020 Mission-Associated Spacecraft Assembly Facility Surfaces—Recommendations for Two New Genera and One Species
Source: J Fungi (Basel). 2022 Dec 23;9(1):31. doi: 10.3390/jof9010031 (PMC9864340; doi:10.3390/jof9010031)
Supplement: Supplementary file 1 [file jof-09-00031-s001.zip › 14. Table S5 Proteomes.pdf]

**Supplemental tables S5:** Proteomics data utilized in developing WGS-based phylogenomic tree					
**Genus**	**Species**	**Strain**	**Version**	**JGI ID**	**Filename**
Aaosphaeria	*arxii*	CBS 175.79	v1.0	Aaoar1	Aaoar1_GeneCatalog_proteins_20140429.aa.fasta.gz
Acidomyces	*richmondensis*	BFW		Aciri1_iso	Aciri1_iso_GeneCatalog_proteins_20111207.aa.fasta.gz
Acrocalymma	*vagum*	DSE3081	v1.0	Acrvag1	Acrvag1_GeneCatalog_proteins_20210226.aa.fasta
Alternaria	*alternata*	ATCC 11680		Alalt1	Alalt1_GeneCatalog_proteins_20190828.aa.fasta
Alternaria	*brassicicola*	ATCC 96836		Albra1	Albra1_GeneCatalog_proteins_20190921.aa.fasta
Aliquandostipite	*khaoyaiensis*	CBS 118232	v1.0	Alikh1	Alikh1_GeneCatalog_proteins_20170216.aa.fasta
Alternaria	*arborescens*	BMP0308		Altar1	Altar1_GeneCatalog_proteins_20190921.aa.fasta
Alternaria	*capsici*	BMP0180		Altca1	Altca1_GeneCatalog_proteins_20190921.aa.fasta
Alternaria	*carthami*	BMP1963		Altcar1	Altcar1_GeneCatalog_proteins_20190921.aa.fasta
Alternaria	*crassa*	BMP0172		Altcr1	Altcr1_GeneCatalog_proteins_20191005.aa.fasta
Alternaria	*dauci*	BMP0167		Altda1	Altda1_GeneCatalog_proteins_20191005.aa.fasta
Alternaria	*fragaria*	BMP3062		Altfr1	Altfr1_GeneCatalog_proteins_20191015.aa.fasta
Alternaria	*gaisen*	BMP2338		Altga1	Altga1_GeneCatalog_proteins_20191016.aa.fasta
Alternaria	*limoniasperae*	BMP2335		Altli1	Altli1_GeneCatalog_proteins_20191016.aa.fasta
Alternaria	*longipes*	BMP0313		Altlo1	Altlo1_GeneCatalog_proteins_20191016.aa.fasta
Alternaria	*macrospora*	BMP1949		Altma1	Altma1_GeneCatalog_proteins_20191016.aa.fasta
Alternaria	*porri*	BMP0178		Altpo1	Altpo1_GeneCatalog_proteins_20191016.aa.fasta
Alternaria	*rosae*	MPI-PUGE-AT-0040	v1.0	Altro1	Altro1_GeneCatalog_proteins_20180320.aa.fasta
Alternaria	*solani*	BMP0185		Altso1	Altso1_GeneCatalog_proteins_20191016.aa.fasta
Alternaria	*sp.*	UNIPAMPA012	v1.0	Altsp012	Altsp012_1_GeneCatalog_proteins_20201206.aa.fasta
Alternaria	*tangelonis*	BMP2327	v1.0	Altta1	Altta1_GeneCatalog_proteins_20191030.aa.fasta
Alternaria	*tenuissima*	BMP0304		Altte1	Altte1_GeneCatalog_proteins_20191025.aa.fasta
Alternaria	*tomatophila*	BMP2032		Alttom1	Alttom1_GeneCatalog_proteins_20200625.aa.fasta
Alternaria	*mali*	BMP3063		Amal3063	Amal3063_GeneCatalog_proteins_20191016.aa.fasta
Amniculicola	*lignicola*	CBS 123094	v1.0	Amnli1	Amnli1_GeneCatalog_proteins_20140304.aa.fasta
Ampelomyces	*quisqualis*	HMLAC05119	v1.0	Ampqui1	Ampqui1_GeneCatalog_proteins_20160127.aa.fasta
Aplosporella	*prunicola*	CBS 121.167	v1.0	Aplpr1	Aplpr1_GeneCatalog_proteins_20121211.aa.fasta
Aquilomyces	*patris*	DSE4099	v1.0	Aqupat1	Aqupat1_GeneCatalog_proteins_20210226.aa.fasta
Ascochyta	*rabiei*	ArDII		Ascra1	Ascra1_GeneCatalog_proteins_20170704.aa.fasta
Aulographum	*hederae*		v2.0	Aulhe2	Aulhe2_GeneCatalog_proteins_20130401.aa.fasta
Aureobasidium	*pullulans*	NBB7.2.1	v1.0	AurpulNBB1	AurpulNBB1_GeneCatalog_proteins_20190122.aa.fasta
Aureobasidium	*pullulans var. melanogenum*	CBS 110374		Aurpu_var_mel1	Aurpu_var_mel1_GeneCatalog_proteins_20130418.aa.fasta
Aureobasidium	*pullulans var. namibiae*	CBS 147.97		Aurpu_var_nam1	Aurpu_var_nam1_GeneCatalog_proteins_20130418.aa.fasta
Aureobasidium	*pullulans var. pullulans*	EXF-150		Aurpu_var_pul1	Aurpu_var_pul1_GeneCatalog_proteins_20130219.aa.fasta
Aureobasidium	*pullulans var. subglaciale*	EXF-2481		Aurpu_var_sub1	Aurpu_var_sub1_GeneCatalog_proteins_20120515.aa.fasta
Baudoinia	*compniacensis*	UAMH 10762 (408982(	v1.0	Bauco1	Bauco1_GeneCatalog_proteins_20110511.aa.fasta
Bimuria	*novae-zelandiae*	CBS 107.79	v1.0	Bimnz1	Bimnz1_GeneCatalog_proteins_20150128.aa.fasta
Boeremia	*exigua*	MPI-SDFR-AT-0100	v1.0	Boeex1	Boeex1_GeneCatalog_proteins_20171129.aa.fasta
Botryosphaeria	*dothidea*			Botdo1	Botdo1_1_GeneCatalog_proteins_20150128.aa.fasta
Byssothecium	*circinans*	CBS 675.92	v1.0	Bysci1	Bysci1_GeneCatalog_proteins_20140627.aa.fasta
Cenococcum	*geophilum*	1.58	v2.0	Cenge3	Cenge3_GeneCatalog_proteins_20130403.aa.fasta
Cercospora	*berteroae*	CBS 538.71		Cerbe1	Cerbe1_GeneCatalog_proteins_20210311.aa.fasta
Cercospora	*zeae-maydis*		v1.0	Cerzm1	Cerzm1_GeneCatalog_proteins_20111029.aa.fasta
Cochliobolus	*heterostrophus*	Hm338	v1.0	ChetHm338	ChetHm338_1_GeneCatalog_proteins_20200308.aa.fasta
Cochliobolus	*heterostrophus*	Hm540		ChetHm540	ChetHm540_1_GeneCatalog_proteins_20210514.aa.fasta
Clavariopsis	*aquatica*	WDA-00-1		Claaq1	Claaq1_GeneCatalog_proteins_20200530.aa.fasta
Clathrospora	*elynae*	CBS 161.51	v1.0	Clael1	Clael1_GeneCatalog_proteins_20140828.aa.fasta
Cladosporium	*fulvum*		v1.0	Clafu1	Clafu1_GeneCatalog_proteins_20110826.aa.fasta
Cladosporium	*sphaerospermum*	UM843		Clasph1	Clasph1_GeneCatalog_proteins_20160727.aa.fasta
Clohesyomyces	*aquaticus*		v1.0	Cloaq1	Cloaq1_GeneCatalog_proteins_20160307.aa.fasta
Cochliobolus	*carbonum*	26-R-13	v1.0	Cocca1	Cocca1_GeneCatalog_proteins_20120427.aa.fasta
Cochliobolus	*heterostrophus*	C5	v3.0	CocheC5	CocheC5_4m_GeneCatalog_proteins_20210518.aa.fasta
Cochliobolus	*lunatus*	m118	v2.0	Coclu2	Coclu2_GeneCatalog_proteins_20120521.aa.fasta
Cochliobolus	*miyabeanus*	ATCC 44560	v1.0	Cocmi1	Cocmi1_GeneCatalog_proteins_20120427.aa.fasta
Cochliobolus	*sativus*	ND90Pr	v1.0	Cocsa1	Cocsa1_GeneCatalog_proteins_20110610.aa.fasta
Cochliobolus	*victoriae*	FI3	v1.0	Cocvi1	Cocvi1_GeneCatalog_proteins_20120501.aa.fasta
Coniosporium	*apollinis*	CBS100218		Conap1	Conap1_GeneCatalog_proteins_20160826.aa.fasta
Corynespora	*cassiicola*	CCP	v1.0	Corca1	Corca1_GeneCatalog_proteins_20140615.aa.fasta
Cryomyces	*antarcticus*	CBS 116301	v3.0	Cryan3	Cryan3_GeneCatalog_proteins_20200914.aa.fasta
Cryomyces	*minteri*	CCFEE 5187		Crymi1	Crymi1_GeneCatalog_proteins_20210419.aa.fasta
Cucurbitaria	*berberidis*	CBS 394.84	v1.0	Cucbe1	Cucbe1_GeneCatalog_proteins_20130102.aa.fasta
Teichospora	*sp.*	FL1471	v1.0	Currey1	Currey1_GeneCatalog_proteins_20180707.aa.fasta
Darksidea	*alpha*	DSE7016	v1.0	Daral1	Daral1_GeneCatalog_proteins_20210113.aa.fasta
Darksidea	*beta*	DSE7011	v1.0	Darbet1	Darbet1_GeneCatalog_proteins_20211018.aa.fasta
Decorospora	*gaudefroyi*		v1.0	Decga1	Decga1_GeneCatalog_proteins_20150425.aa.fasta
Delitschia	*confertaspora*	ATCC 74209	v1.0	Delco1	Delco1_GeneCatalog_proteins_20140624.aa.fasta
Valsariales	*sp.*	FL0756	v1.0	DelFL0756	DelFL0756_1_GeneCatalog_proteins_20181025.aa.fasta
Delphinella	*strobiligena*	CBS 735.71	v1.0	Delst1	Delst1_GeneCatalog_proteins_20160906.aa.fasta
Dendryphion	*nanum*	MPI-CAGE-CH-0243	v1.0	Denna1	Denna1_GeneCatalog_proteins_20180321.aa.fasta
Didymella	*exigua*	CBS 183.55	v1.0	Didex1	Didex1_GeneCatalog_proteins_20120924.aa.fasta
Didymella	*zeae-maydis*	3018		Didma1	Didma1_GeneCatalog_proteins_20160126.aa.fasta
Didymocrea	*sadasivanii*	CBS 438.65	v1.0	Didsa1	Didsa1_GeneCatalog_proteins_20170324.aa.fasta

Diplodia	*seriata*	DS831		Dipse1	Dipse1_GeneCatalog_proteins_20150930.aa.fasta
Dissoconium	*aciculare*		v1.0	Disac1	Disac1_GeneCatalog_proteins_20130805.aa.fasta
Dothideomycetes	*sp.*	P12C66	v1.0	DotP12C66	DotP12C66_GeneCatalog_proteins_20201103.aa.fasta
Dothistroma	*septosporum*	NZE10	v1.0	Dotse1	Dotse1_GeneCatalog_proteins_20100818.aa.fasta
Dothidotthia	*symphoricarpi*		v1.0	Dotsy1	Dotsy1_GeneCatalog_proteins_20131210.aa.fasta
Elsinoe	*ampelina*	CECT20119	v1.0	Elsamp1	Elsamp1_GeneCatalog_proteins_20151205.aa.fasta
Epicoccum	*nigrum*	cont 1108929	v1.0	Epini1	Epini1_GeneCatalog_proteins_20190403.aa.fasta
Eremomyces	*bilateralis*	CBS 781.70	v1.0	Erebi1	Erebi1_GeneCatalog_proteins_20150104.aa.fasta
Fenestella	*fenestrata*	ATCC 66461	v1.0	Fenfe1	Fenfe1_GeneCatalog_proteins_20180419.aa.fasta
Flavomyces	*fulophazii*	DSE8309	v1.0	Flaful1	Flaful1_GeneCatalog_proteins_20210226.aa.fasta
Friedmanniomyces	*endolithicus*	CCFEE 5311		Frien1	Frien1_GeneCatalog_proteins_20210426.aa.fasta
Friedmanniomyces	*simplex*	CCFEE 5184		Frisi1	Frisi1_GeneCatalog_proteins_20210419.aa.fasta
Gloniopsis	*sp.*	FL0384	v1.0	Glonio1	Glonio1_GeneCatalog_proteins_20180712.aa.fasta
Glonium	*stellatum*	CBS 207.34	v1.0	Glost2	Glost2_GeneCatalog_proteins_20140303.aa.fasta
Herpotrichia	*sp.*	AK1299	v1.0	Herpot1	Herpot1_1_GeneCatalog_proteins_20180323.aa.fasta
Hortaea	*acidophila*	CBS 113389	v1.0	Horac1	Horac1_GeneCatalog_proteins_20160326.aa.fasta
Hortaea	*thailandica*	CCFEE 6315		Horth1	Horth1_GeneCatalog_proteins_20210419.aa.fasta
Hortaea	*werneckii*	EXF-2000M0	v1.0	Horwer1	Horwer1_GeneCatalog_proteins_20160803.aa.fasta
Hysterium	*pulicare*			Hyspu1	Hyspu1_1_GeneCatalog_proteins_20150205.aa.fasta
Jahnula	*aquatica*	CBS 123560	v1.0	Jahaq1	Jahaq1_GeneCatalog_proteins_20181114.aa.fasta
Karstenula	*rhodostoma*	CBS 690.94	v1.0	Karrh1	Karrh1_GeneCatalog_proteins_20140225.aa.fasta
Lentithecium	*fluviatile*		v1.0	Lenfl1	Lenfl1_GeneCatalog_proteins_20121217.aa.fasta
Leptosphaeria	*microscopica*	UNIPAMPA013	v1.0	Lepmi1	Lepmi1_GeneCatalog_proteins_20210729.aa.fasta
Leptosphaeria	*maculans*			Lepmu1	Lepmu1_GeneCatalog_proteins_20110301.aa.fasta
Lepidopterella	*palustris*		v1.0	Leppa1	Leppa1_GeneCatalog_proteins_20130322.aa.fasta
Lindgomyces	*ingoldianus*	ATCC 200398	v1.0	Linin1	Linin1_GeneCatalog_proteins_20141206.aa.fasta
Lineolata	*rhizophorae*	ATCC 16933	v1.0	Linrh1	Linrh1_GeneCatalog_proteins_20160326.aa.fasta
Lizonia	*empirigonia*	CBS 542.76	v1.0	Lizem1	Lizem1_GeneCatalog_proteins_20150430.aa.fasta
Mytilinidiaceae	*sp.*	NC0267	v1.0	Lophiu1	Lophiu1_GeneCatalog_proteins_20181114.aa.fasta
Lophiostoma	*macrostomum*		v1.0	Lopma1	Lopma1_GeneCatalog_proteins_20130805.aa.fasta
Lophium	*mytilinum*	CBS 269.34	v1.0	Lopmy1	Lopmy1_GeneCatalog_proteins_20140604.aa.fasta
Lophiotrema	*nucula*	CBS 627.86	v1.0	Lopnu1	Lopnu1_GeneCatalog_proteins_20160906.aa.fasta
Macroventuria	*anomochaeta*	CBS 525.71	v1.0	Macan1	Macan1_GeneCatalog_proteins_20140424.aa.fasta
Macrophomina	*phaseolina*	MS6		Macph1	Macph1_GeneCatalog_proteins_20131211.aa.fasta
Macrophomina	*phaseolina*	MPI-SDFR-AT-0080	v1.0	Macpha1	Macpha1_GeneCatalog_proteins_20180322.aa.fasta
Massarina	*eburnea*	CBS 473.64	v1.0	Maseb1	Maseb1_GeneCatalog_proteins_20141122.aa.fasta
Massariosphaeria	*phaeospora*	CBS 611.86	v1.0	Masph1	Masph1_GeneCatalog_proteins_20160922.aa.fasta
Melanomma	*pulvis-pyrius*		v1.0	Melpu1	Melpu1_GeneCatalog_proteins_20130415.aa.fasta
Melanops	*tulasnei*	CBS 116805	v1.0	Meltu1	Meltu1_GeneCatalog_proteins_20180421.aa.fasta
Pseudocercospora	*fijiensis*		v2.0	Mfijiensis	Mfijiensis_v2.FrozenGeneCatalog_20100402.proteins.fasta
Mycosphaerella	*graminicola*		v2.0	Mgraminicolav2.Fro	Mgraminicolav2.FrozenGeneCatalog20080910.proteins.fasta
Microthyrium	*microscopicum*	CBS 115976	v1.0	Micmi1	Micmi1_GeneCatalog_proteins_20150625.aa.fasta
Microdiplodia	*sp.*	AK1800	v1.0	Microd1	Microd1_GeneCatalog_proteins_20180925.aa.fasta
Mycosphaerella	*eumusae*	CBS 114824		Myceu1	Myceu1_GeneCatalog_proteins_20210311.aa.fasta
Myriangium	*duriaei*	CBS260.36	v1.0	Myrdu1	Myrdu1_GeneCatalog_proteins_20121211.aa.fasta
Myriangiaceae	*sp.*	NC1570	v1.0	Myrian1	Myrian1_GeneCatalog_proteins_20181114.aa.fasta
Mytilinidion	*resinicola*	CBS304.34	v1.0	Mytre1	Mytre1_GeneCatalog_proteins_20150115.aa.fasta
Neofusicoccum	*parvum*	UCRNP2		Neopa1	Neopa1_GeneCatalog_proteins_20130909.aa.fasta
Ophiobolus	*disseminans*	CBS113818	v1.0	Ophdi1	Ophdi1_GeneCatalog_proteins_20140614.aa.fasta
Paraphoma	*chrysanthemicola*	MPI-GEGE-AT-0034	v1.0	Parch1	Parch1_GeneCatalog_proteins_20171014.aa.fasta
Parafenestella	*ontariensis*	EI-6	v1.0	Paront1	Paront1_GeneCatalog_proteins_20220408.aa.fasta
Paraconiothyrium	*sporulosum*	AP3s5-JAC2a	v1.0	Parsp1	Parsp1_GeneCatalog_proteins_20141011.aa.fasta
Patellaria	*atrata*		v1.0	Patat1	Patat1_GeneCatalog_proteins_20130514.aa.fasta
Phyllosticta	*citricarpa*	CBS 102373	v1.0	Pcit120373	Pcit120373_GeneCatalog_proteins_20200613.aa.fasta
Phyllosticta	*citrichinaensis*	CBS 129764	v1.0	Pcit129764	Pcit129764_GeneCatalog_proteins_20200914.aa.fasta
Phyllosticta	*citriasiana*	CBS 120426	v2.0	Pcitr2	Pcitr2_GeneCatalog_proteins_20200618.aa.fasta
Periconia	*macrospinosa*	DSE2036	v1.0	Perma1	Perma1_GeneCatalog_proteins_20141217.aa.fasta
Phaeosphaeriaceae	*sp.*	PMI_808	v1.0	PhaPMI808	PhaPMI808_GeneCatalog_proteins_20160324.aa.fasta
Phaeosphaeria	*poagena*	MPI-PUGE-AT-0046c	v1.0	Phapo1	Phapo1_GeneCatalog_proteins_20180321.aa.fasta
Phyllosticta	*capitalensis*	CBS 111638	v2.0	Phcapit2	Phcapit2_GeneCatalog_proteins_20200621.aa.fasta
Phyllosticta	*citribraziliensis*	CBS 100098	v1.0	Phcit1	Phcit1_GeneCatalog_proteins_20161221.aa.fasta
Phoma	*multirostrata*	7a	v1.0	Phomu1	Phomu1_GeneCatalog_proteins_20180815.aa.fasta
Phoma	*tracheiphila*	IPT5	v1.0	Photr1	Photr1_GeneCatalog_proteins_20140424.aa.fasta
Phyllosticta	*paracitricarpa*	CBS 141357	v1.0	Phy27169	Phy27169_GeneCatalog_proteins_20170510.aa.fasta
Phyllosticta	*sp.*	CPC27913	v1.0	Phycpc1	Phycpc1_GeneCatalog_proteins_20170406.aa.fasta
Piedraia	*hortae*	CBS 480.64	v1.1	Pieho1	Pieho1_1_GeneCatalog_proteins_20130807.aa.fasta
Pleospora	*avicennia*	ATCC 66911	v1.0	Pleav1	Pleav1_GeneCatalog_proteins_20180620.aa.fasta
Pleosporaceae	*sp.*	PMI_138	v1.0	PlePMI138	PlePMI138_1_GeneCatalog_proteins_20190917.aa.fasta
Pleomassaria	*siparia*		v1.0	Plesi1	Plesi1_GeneCatalog_proteins_20130603.aa.fasta
Polychaeton	*citri*		v1.0	Polci1	Polci1_GeneCatalog_proteins_20130311.aa.fasta
Polyplosphaeria	*fusca*	CBS 125425	v1.0	Polfu1	Polfu1_GeneCatalog_proteins_20140813.aa.fasta
Pseudofusicoccum	*sp.*	LGMF1611	v1.0	Pse1611	Pse1611_1_GeneCatalog_proteins_20201211.aa.fasta
Pseudovirgaria	*hyperparasitica*	CBS121739	v1.0	Psehy1	Psehy1_GeneCatalog_proteins_20140829.aa.fasta
Pseudocercospora	*musae*	CBS 116634		Psemus1	Psemus1_GeneCatalog_proteins_20210311.aa.fasta

Pseudocercospora	*ulei*	ERN8	v1.0	Pseule1	Pseule1_GeneCatalog_proteins_20190530.aa.fasta
Pyrenochaeta	*inflorescentiae*	CORFU0001	v1.0	Pyrinf1	Pyrinf1_GeneCatalog_proteins_20200511.aa.fasta
Pyrenochaeta	*lycopersici*	MPI-SDFR-AT-0127	v1.0	Pyrly1	Pyrly1_GeneCatalog_proteins_20170623.aa.fasta
Pyrenochaeta	*sp.*	DS3sAY3a	v1.0	Pyrsp1	Pyrsp1_GeneCatalog_proteins_20141006.aa.fasta
Pyrenophora	*teres f. teres*			Pyrtt1	Pyrtt1_GeneCatalog_proteins_20110408.aa.fasta
Rachicladosporium	*sp.*	CCFEE 5018		Rac5018	Rac5018_1_GeneCatalog_proteins_20210311.aa.fasta
Rachicladosporium	*antarcticum*	CCFEE 5527		Racan1	Racan1_GeneCatalog_proteins_20210311.aa.fasta
Rhizodiscina	*lignyota*	CBS 133067	v1.0	Rhili1	Rhili1_GeneCatalog_proteins_20150422.aa.fasta
Rhytidhysteron	*rufulum*			Rhyru1	Rhyru1_1_GeneCatalog_proteins_20150204.aa.fasta
Saccharata	*proteae*	CBS 121410	v1.0	Sacpr1	Sacpr1_GeneCatalog_proteins_20140222.aa.fasta
Septoria	*musiva*	SO2202	v1.0	Sepmu1	Sepmu1_GeneCatalog_proteins_20100915.aa.fasta
Septoria	*populicola*		v1.0	Seppo1	Seppo1_GeneCatalog_proteins_20110720.aa.fasta
Setomelanomma	*holmii*	CBS 110217	v1.0	Setho1	Setho1_GeneCatalog_proteins_20150424.aa.fasta
Setosphaeria	*turcica*	Et28A	v2.0	Settu3	Settu3_GeneCatalog_proteins_20170818.aa.fasta
Sporormia	*fimetaria*		v1.0	Spofi1	Spofi1_GeneCatalog_proteins_20130818.aa.fasta
Stagonospora	*nodorum*	SN15	v2.0	Stano2	Stano2_GeneCatalog_proteins_20110506.aa.fasta
Stagonospora	*sp.*	SRC1lsM3a	v1.0	Stasp1	Stasp1_GeneCatalog_proteins_20141004.aa.fasta
Stemphylium	*lycopersici*	CIDEFI-216		Stely1	Stely1_GeneCatalog_proteins_20170711.aa.fasta
Stomiopeltis	*betulae*	CBS 114420	v1.0	Stobe1	Stobe1_GeneCatalog_proteins_20180620.aa.fasta
Teratosphaeriacea	*sp.*	NC1134	v1.0	TerNC1134	TerNC1134_1_GeneCatalog_proteins_20190222.aa.fasta
Teratosphaeria	*nubilosa*	CBS 116005	v1.0	Ternu1	Ternu1_GeneCatalog_proteins_20140925.aa.fasta
Tothia	*fuscella*	CBS 130266	v1.0	Totfu1	Totfu1_GeneCatalog_proteins_20140831.aa.fasta
Trematosphaeria	*pertusa*	CBS 122368	v1.0	Trepe1	Trepe1_GeneCatalog_proteins_20140415.aa.fasta
Trichodelitschia	*bisporula*	CBS 262.69	v1.0	Tribi1	Tribi1_GeneCatalog_proteins_20141117.aa.fasta
Viridothelium	*virens*		v1.0	Tryvi1	Tryvi1_GeneCatalog_proteins_20130617.aa.fasta
Venturia	*effusa*			Veneff1	Veneff1_GeneCatalog_proteins_20200529.aa.fasta
Venturia	*inaequalis*			Venin1	Venin1_GeneCatalog_proteins_20141010.aa.fasta
Venturia	*pirina*			Venpi1	Venpi1_GeneCatalog_proteins_20150407.aa.fasta
Venturia	*populina*	CBS 256.38	v1.0	Venpo1	Venpo1_GeneCatalog_proteins_20220406.aa.fasta
Venturiales	*sp.*	P10P74	v1.0	VenspP74	VenspP74_1_GeneCatalog_proteins_20201207.aa.fasta
Verruculina	*enalia*	CBS 304.66	v1.0	Veren1	Veren1_GeneCatalog_proteins_20140626.aa.fasta
Verruconis	*gallopava*			Verga1	Verga1_GeneCatalog_proteins_20160827.aa.fasta
Westerdykella	*ornata*	CBS 379.55	v1.0	Wesor1	Wesor1_GeneCatalog_proteins_20140626.aa.fasta
Zasmidium	*cellare*	ATCC 36951	v1.0	Zasce1	Zasce1_GeneCatalog_proteins_20120808.aa.fasta
Zopfia	*rhizophila*		v1.0	Zoprh1	Zoprh1_GeneCatalog_proteins_20130606.aa.fasta
Zymoseptoria	*ardabiliae*	STIR04_1.1.1		Zymar1	Zymar1_GeneCatalog_proteins_20141012.aa.fasta
Zymoseptoria	*brevis*	Zb18110		Zymbr1	Zymbr1_GeneCatalog_proteins_20170711.aa.fasta
Zymoseptoria	*pseudotritici*	STIR04_2.2.1		Zymps1	Zymps1_GeneCatalog_proteins_20141012.aa.fasta
